# Exploring the Role of Active Functional Microbiota in Flavor Generation by Integrated Metatranscriptomics and Metabolomics during Niulanshan Baijiu Fermentation

**DOI:** 10.3390/foods12224140

**Published:** 2023-11-15

**Authors:** Yuanyuan Pan, Ying Wang, Wenjun Hao, Sen Zhou, Chengbao Duan, Qiushi Li, Jinwang Wei, Gang Liu

**Affiliations:** 1 State Key Laboratory of Mycology, Institute of Microbiology, Chinese Academy of Sciences, Beijing 100101, China; panyy@im.ac.cn (Y.P.); 18813111804@163.com (C.D.); liqiushi192@mails.ucas.ac.cn (Q.L.); 2 Niulanshan Distillery, Beijing Shunxin Agriculture Company Limited, Beijing 101301, China; wangying.2006h@163.com (Y.W.); hwj1983hwj@126.com (W.H.); ak19861118ka@126.com (S.Z.); 3 University of Chinese Academy of Sciences, Beijing 100049, China

**Keywords:** light-flavor baijiu, metatranscriptomics, active functional microbiota, untargeted metabolomics, flavor generation, fermented grain

## Abstract

Active functional microbiota for producing volatile flavors is critical to Chinese baijiu fermentation. Microbial communities correlated with the volatile metabolites are generally explored using DNA-based sequencing and metabolic analysis. However, the active functional microbiota related to the volatile flavor compounds is poorly understood. In this study, an integrated metatranscriptomic and metabolomics analysis was employed to unravel the metabolite profiles comprehensively and the contributing active functional microbiota for flavor generation during Niulanshan baijiu fermentation. A total of 395, 83, and 181 compounds were annotated using untargeted metabolomics, including LC-MS, GC-MS, and HS-SPME-GC-MS, respectively. Significant variances were displayed in the composition of compounds among different time-point samples according to the heatmaps and orthogonal partial least-square discriminant analysis. The correlation between the active microbiota and the volatile flavors was analyzed based on the bidirectional orthogonal partial least squares discriminant analysis (O2PLS-DA) model. Six bacterial genera, including *Streptococcus*, *Lactobacillus*, *Pediococcus*, *Campylobacter*, *Yersinia,* and *Weissella*, and five fungal genera of *Talaromyces*, *Aspergillus*, *Mixia*, *Rhizophagus,* and *Gloeophyllum* were identified as the active functional microbiota for producing the volatile flavors. In summary, this study revealed the active functional microbial basis of unique flavor formation and provided novel insights into the optimization of Niulanshan baijiu fermentation.

## 1. Introduction

Baijiu is a typical representative of Chinese traditional fermented food and is regarded as one of the six well-known distilled spirits in the world [1,2]. Chinese baijiu is brewed using solid-state fermentation with high-quality sorghum or other grains as raw materials. Jiuqu, such as Daqu, Xiaoqu, or Fuqu, where prokaryotes and eukaryotes cohabit, interact, and communicate, is used as the starter for saccharification and fermentation of raw materials, and a series of complicated enzymatic, organic chemical and biochemical reactions proceed through the aid of the microbial community in open fermentation environments, followed by distillation, blending, and storage to form the final products [3,4]. The flavor components of baijiu mainly come from the aroma of the raw materials, substances produced during fermentation, distillation of fermented grains, and microbial metabolites from starter and fermented containers [5]. There are over 2000 compounds detected in baijiu, including alcohols, organic acids, esters, aldehydes, ketones, amino acids, acetals, furans, hydroxyl compounds, nitrogen compounds, and sulfur-containing compounds, which endow baijiu with unique flavor and taste [6,7]. According to the composition and proportion of flavor compounds, Chinese baijiu is divided into 12 types of flavor, of which jiang-flavor (Maotai-flavor), strong-flavor (Luzhou-flavor), and light-flavor are the three most important representatives.

Niulanshan baijiu (NLS), as a famous brand and national cultural heritage in China, is a representative of light-flavor baijiu (LFB) and is popular among consumers for its purity, pleasantly fruity, mild taste, and refreshing aftertaste [8]. The baijiu flavor formation is mainly dependent on its unique fermentation process, distinct starter, carefully selected raw materials, and special fermentation workshops. NLS adopts Erguotou-producing craft and is brewed by using high-quality wheat, barley, and peas to make low-temperature Daqu. Sorghum from Northeast China is used as the raw material, and an earthen jar (Digang) is used as the classic fermentation container [9]. Much progress has been achieved in the fermentation process and flavor generation of NLS in recent years. For example, four unclassified bacteria were unique, and the ratio of fungi to bacteria was the highest in Niulanshan Daqu when compared with other aroma-type Daqu, including Wuling Daqu (jiang-flavor), Baisha Daqu (mixed-flavor) and Deshan Daqu (strong-flavor) [10]. In addition, the composition, drivers, and functions of the active microorganisms in the fermented grains were revealed by metatranscriptomics systematically, which was different from reports about the dominant microorganisms during fermentation [11]. Moreover, the phylogeny and population structure of the amylolytic yeast *Saccharomycopsis fibuligera* from Niulanshan Distillery were analyzed, and these strains were distributed in the same lineage from the fermented grains and low-temperature Daqu [12]. Meanwhile, a total of fifty nine odorants were found in NLS samples at different storage years, and five of them were first identified as aroma-active compounds in LFB [9].

Among all the mentioned fermentation parameters, fermented grains are the main carrier of microbial fermentation and the direct contributors to baijiu flavor [13]. Therefore, fermented grains, where microbial communities play a vital role, and flavors are formed, have always been the focus of baijiu fermentation research. Based on metagenomics and metabolomics analysis of the fermented grains from Fenjiu Distillery Co., Ltd., the correlation between microorganisms and their function was established [8]. Additionally, the effects of fermentation periods on the flavor components of light-flavor Xiaoqu baijiu were studied by HS-SPME-GC-MS. The esters and alcohols, including ethyl acetate, diethyl succinate, phenylethyl alcohol, ethyl hydrogen succite, and n-propanol, accumulated to the highest abundance after 98 days of fermentation. In contrast to the normal fermented grains, the content of n-propanol was increased significantly in the ultra-long fermentation samples [14].

Our previous study based on metatranscriptomics clarified the active microbial composition, drivers, and their functions in NLS fermentation. However, the dynamic change in the metabolites and the active functional microbiota contributed to the volatile flavors in the fermented grains are poorly understood, generating a mystery of NLS flavor formation. In this study, integrated metatranscriptomics and untargeted metabolomics were employed to elucidate the dynamic changes in metabolites and the potential correlation between the active microbiota and the volatile flavors based on the O2PLS-DA model. Furthermore, the active functional microbiota involved in the production of the volatile flavors was identified. Herein, we provided an overview of the comprehensive metabolite compositional variety and the contributed active functional microbiota in the fermented grains in the hope of facilitating a better understanding of flavor formation and providing the basis to improve the quality of NLS.

## 2. Materials and Methods

### 2.1. Sampling of the Fermented Grains

The fermented grains were collected from Niulanshan Distillery (Beijing, China) in November 2019. For metatranscriptomic analysis, sampling collection was performed as previously described [11]. For metabolomic analysis, two samples from each of the three different earthen jars were collected at each time point. The samples from earthen jars A, B, and C were collected at 0 (D0) and 8 (D8) days of fermentation, respectively. The samples from earthen jars D, E, and F were collected at 6 (D6), 16 (D16), and 27 (D27) days of fermentation, respectively. Jar G was used to fill the space left in the above jars after sampling. A total of 30 samples from 5 time points were collected. The sampling schematic diagram for metabolomic analysis is shown in Figure S1. All samples were collected quickly and stored in a freezer at −70 °C prior to measurement. Six biological replicates were performed at each time point.

### 2.2. Metatranscriptomic Sequencing

RNA extraction was performed as previously described [11]. After the integrity and quality of RNA were assessed, equivalent RNAs (5 μg) were used for cDNA library construction. Firstly, rRNA was depleted according to the manuscript of the Ribo-Zero™ rRNA Removal Kit (Illumina, San Diego, CA, USA), and the remaining RNAs were fragmented and reverse transcribed to cDNA. Then, cDNA was synthesized to U-labeled second-stranded DNA. Subsequently, the addition of A-base and ligation with adaptor were accompanied. Finally, U-labeled second-stranded DNA was digested with UDG enzyme, and 300 bp (±50 bp) PCR products were sequenced on an IlluminaHiseq 4000 at the LC Sciences (Hangzhou, China).

### 2.3. Metabolite Profiles by LC-MS Analysis

A total of 100 mg of fermented grains was mixed with 600 μL of 2-chlorophenylalanine (4 ppm) methanol (−20 °C) and vortexed for 30 sec. The mixture was ground by a high-throughput tissue grinder for 90 sec at 60 Hz and then sonicated for 10 min. After centrifuging, the supernatant was filtered. A total of 20 μL of each sample was taken as the quality control (QC) sample, and the remainder was used for LC-MS detection. LC-MS analysis was performed as previously described [15,16].

### 2.4. Metabolite Profiles by GC-MS Analysis

For GC-MS analysis, 100 mg of samples was mixed with 500 μL of ddH_2_O and vortexed for 60 sec. To the mixture, 1 mL of pre-cooled methanol and 60 μL of heptadecanoic acid (0.2 mg/mL stock in methanol, as the internal standard) were added, ultrasonicated for 30 min and then cooled on ice. Subsequently, the samples were centrifuged, and the supernatant was concentrated through drying. A total of 60 μL of 15 mg/mL methoxyamine pyridine solution was added into the concentrated samples, vortexed for 30 sec, and then reacted at 37 °C for 120 min. A total of 60 μL BSTFA reagent (containing 1% TMCS) was added to the mixture and left to sit for 90 min. After centrifuging, 20 μL of supernatant from each sample was used as the QC sample. The rest of the samples were used for GC-MS detection. Agilent 7890A/5975C GC-MS system (Agilent, Santa Clara, CA, USA) was used for GC-MS and performed as previously described [17].

### 2.5. Metabolite Profiles by HS-SPME-GC-MS Analysis

A total of 1.5 g of samples were transferred to a 20 mL screwcap vial and preheated at 50 °C, with agitation at 250 rpm for 15 min. The volatile compounds were extracted with 50/30 μm DVB/CAR on PDMS (Supelco, Bellefonte, PA, USA). The analysis was performed using a 7890B-5977B GC/MS system (Agilent, Santa Clara, CA, USA) equipped with a CTC trinity autosampler. Volatile compounds were analyzed and identified as previously described [15,18].

### 2.6. Data Processing

Raw data were first converted to mzXML format using the Proteowizard software package (v3.0.8789) and processed using XCMS running under R (V3.3.2) for peak identification, filtration, and alignment. A data matrix, including m/z, retention time, and intensity, was obtained. The metabolites were identified using the databases of Metlin, MoNA, and Panomic’s own standard database (Suzhou BioNovoGene Biomedical Tech Co., Ltd., Suzhou, China).

### 2.7. Multivariate Statistical Analysis

Normalized data were uploaded for multivariate statistical analysis, including principal components analysis (PCA) and orthogonal partial least-square discriminant analysis (OPLS-DA). To reduce the influence of major metabolites, Pareto scaling was applied to all datasets. The descriptive performance of the OPLS-DA models was determined using R2X and R2Y, while their prediction performance was measured using Q2. The O2PLS-DA model was used to estimate the relationship between the key microorganisms and the volatile metabolites, which consisted of the projection of both the microorganisms (X) and the volatile metabolites (Y) during baijiu fermentation in R via OmicsPLS package [19]. The correlation matrix displayed the pair-wise correlation between microorganisms and the volatile metabolites. Microbial genera and species with variable importance in the projection (VIP) value > 1.0 were regarded as the most important indexes to explain the metabolites. The significance of the correlation coefficient was calculated using the OmicStudio tools at https://www.omicstudio.cn/tool (accessed on 17 June 2023). A high correlation coefficient (|rho| > 0.8, *p* < 0.05) between the microbial genera/species (VIP > 1) and the key volatile compounds were visualized.

## 3. Results

### 3.1. Active Microbial Community Diversity and Composition

Taxonomic annotation of the active microbial community at the superkingdom level indicated that the relative abundance of bacteria and fungi was 40.83% and 40.31%, respectively, at the beginning of fermentation. The relative abundance of bacteria gradually increased to 46.23%, and that of fungi decreased to 25.30% on the 16th day of fermentation. Subsequently, the relative abundance of bacteria and fungi reached 45.40% and 29.55% at the end of fermentation, respectively. In addition to the bacteria and fungi, the relative abundance of archaea and viruses accounted for 0.0036–0.0068% and 0.056–0.15%, respectively, during the fermentation (Figure S2). According to the microbial abundance analysis, the active microbial community with relative abundances higher than 0.1% or 1% at the genus and species level were picked and analyzed (Table S1). When the relative abundance higher than 0.1% was selected, there were 39 genera and 59 species in at least one sample at the beginning of fermentation, then the numbers gradually decreased, and only 21 genera and 37 species existed until the 8th day of fermentation. There were 26 genera and 45 species when the fermentation finished. When a relative abundance higher than 1% was selected, a total of 14 genera and 19 species in at least one sample at the beginning of fermentation, then the numbers dropped to 9 genera and 12 species at day 6 of fermentation and remained stable until the end of fermentation. These results indicated that the diversity of the microbial community decreased along with the fermentation process.

**Figure 1:**
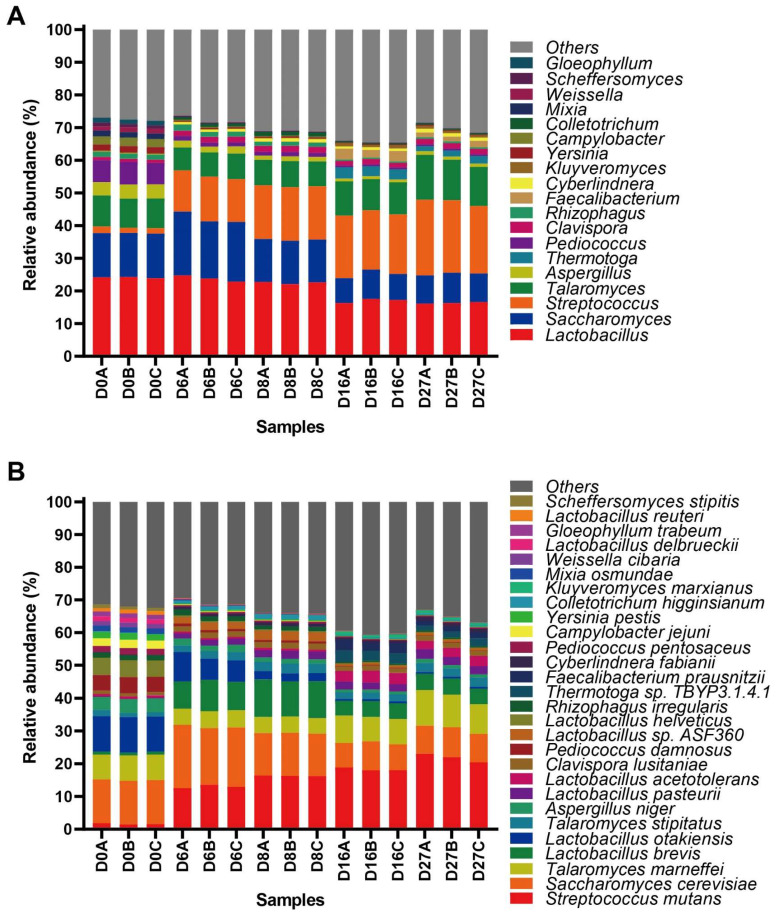
Taxonomic annotation of the active microbial community during NLS fermentation. (**A**) Distribution of the active microbial community with a relative abundance higher than 1% at the genus level. (**B**) Distribution of the active microbial community with a relative abundance higher than 1% at the species level.

A previous study reported that a total of 421 genera were annotated during NLS fermentation by metatranscriptomic analysis [11]. In this study, the microbial community at the genus and species level with a relative abundance higher than 1%, which included 19 genera and 28 species, was comprehensively analyzed. At the beginning of fermentation, the fungal genera, including *Saccharomyces*, *Talaromyces,* and *Aspergillus*, and the bacterial genera, including *Lactobacillus*, *Pediococcus*, *Streptococcus,* and *Campylobacter,* constituted the active predominant genera. Then, the relative abundances of *Streptococcus*, *Saccharomyces*, *Clavispora,* and *Colletotrichum* were further increased and accompanied by a sharp decline in *Aspergillus*, *Pediococcus,* and *Campylobacter* at days 6 and 8 of fermentation. In addition to the genera of *Saccharomyces*, *Talaromyces*, *Lactobacillus,* and *Streptococcus,* which were the active predominant genera, there was a dramatic increase in *Faecalibacterium* and *Thermotoga* at the end of fermentation (Figure 1A).

There were 885 species annotated during NLS fermentation using metatranscriptomic analysis. Species *S. cerevisiae*, *L. otakiensis*, *T. marneffei*, *L. helveticus*, *P. damnosus,* and *A. niger* were dominant at the beginning of fermentation. As the fermentation progresses, the relative abundance of *S. mutans*, *L. brevis*, *L. pasteurii*, *Lactobacillus* sp. ASF360 and *C. lusitaniae* increased sharply, and the abundance of *L. otakiensis*, *T. marneffei*, *L. helveticus*, *P. damnosus,* and *A. niger* decreased simultaneously on day 8 of fermentation. The late stage of fermentation was characterized by the dramatic increase in *F. prausnitzii*, *Thermotoga* sp. TBYP3.1.4.1 and *L. acetotolerans*. When the fermentation proceeded to the late stage, the increase in acids and alcohol inhibited the growth of other *Lactobacillus* with weak acid and alcohol tolerance, resulting in the accumulation of *L. acetotolerans* (Figure 1B). Our results indicated that the active microbiota displayed a dynamic change in NLS fermentation.

### 3.2. Characterization of the Polar Metabolites Based on LC-MS

**Figure 2:**
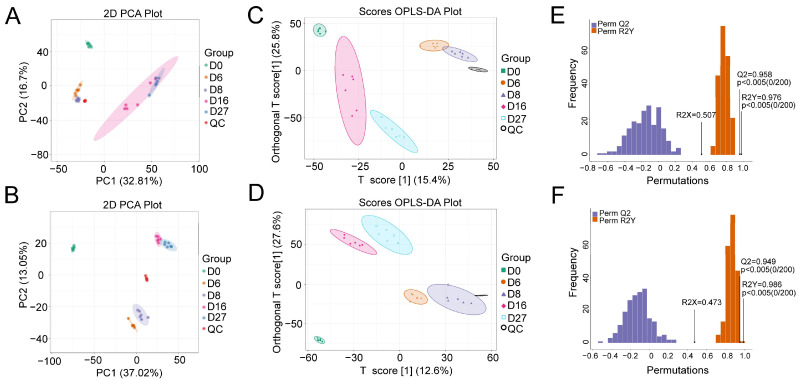
Principle component analysis (PCA) and orthogonal partial least-squares discriminant analysis (OPLS-DA) of the compounds based on the LC-MS detection. (**A**) The PCA model in negative ion mode. (**B**) The PCA model in positive ion mode. (**C**) The OPLS-DA score plot of the different fermentation samples in negative ion mode. (**D**) The OPLS-DA score plot of the different fermentation samples in positive ion mode. (**E**) The 200× permutation tests of the different fermentation samples in negative ion mode. (**F**) The 200× permutation tests of the different fermentation samples in positive ion mode. D0, D6, D8, D16, and D27 represent the fermentation samples collected on 0, 6, 8, 16, and 27 days, respectively.

The metabolite profiles of polar compounds were investigated using a global untargeted metabolomic platform with LC-MS. A total of 10,864 and 6677 compounds were extracted in the positive and negative ionization modes, respectively. Distinct cluster distribution between all detected samples and QC samples was shown in the PCA score plots, indicating that the analytical process was reproducible. Except for the samples of days 16 and 27, which displayed in the negative (Figure 2A) or the positive mode (Figure 2B), other samples were assigned to separate groups according to the fermentation time, implying that dynamic change in the metabolites was taken place during fermentation. The NLS fermentation is divided into three stages, including early stage (0 to 6 days), middle stage (6 to 12 days), and late stage (12 to 28 days), according to the previous reports [11]. Therefore, we postulated that the similarity of metabolites between 16 and 27 days gave the overlapping of two groups in PCA.

When a supervised OPLS-DA was performed, all groups displayed significant separation. The OPLS-DA model was validated using k-fold cross-validation (k = 7). The explained variation R2X (0.507) and R2Y (0.976) was obtained, and the predictive capability Q2 was 0.958 in the negative ionization mode (Figure 2C). In the positive ionization mode, the obtained R2X, R2Y, and Q2 were 0.473, 0.986, and 0.949, respectively, which indicated that the model had good explanatory and predictive ability (Figure 2D). Meanwhile, the permutation test (*n* = 200) was performed to evaluate whether the discriminant model was overfitting. The *p* values of Q2 and R2Y were all lower than 0.005, implying that the models were statistically valid without overfitting (Figure 2E,F).

A total of 395 compounds were annotated and classified into nine categories, including amino acids (147, 37.22%), carbohydrates (66, 16.71%), lipids (60, 15.19%), xenobiotics (45, 11.39%), cofactors and vitamins (35, 8.86%), nucleotides (27, 6.84%), energy (6, 1.52%), peptides (1, 0.25%), and unclassified compounds (8, 2.03%) according to the KEGG and metabolon database (Table S2). Subsequently, hierarchical cluster analysis (HCA) was applied to elucidate the relationship between the compounds and concentrations (Figure S3). The color from sky blue to pink indicated that the relative intensity increased in the heatmap. Based on HCA, all compounds were clearly classified into four clusters. Cluster I contained 50 compounds whose concentrations were significantly increased after 16 and 27 days of fermentation. Cluster I was composed of 19 amino acids, polyols such as vanylglycol and abscisic alcohol, 1 alditol (mannitol), 4 aldehydes (4-aminobutyraldehyde, L-glutamic gamma-semialdehyde, adipate semialdehyde and 2-hydroxybenzaldehyde) and 20 organic acids (L-lactic acid, 2-hydroxybutyric acid, 4-fumarylacetoacetate, etc). Cluster II consisted of 107 compounds with a gradually increasing pattern of concentration during fermentation. Cluster II contained 38 organic acids such as oxoglutaric acid, nicotinic acid and alpha-linolenic acid, D-phenyllactic acid, aldehydes such as benzaldehyde, 3-hydroxybenzaldehyde and 4-methylbenzaldehyde, 22 amino acids, several polyols and alditols such as 2-phenylethanol, erythritol, D-xylitol, tryptophanol, and maltol, etc. We postulated that the compounds in Cluster I and II had a strong correlation with the flavor generation of NLS. The 69 compounds in Cluster III showed the opposite trend, in which concentrations began to decrease after 16 and 27 days of fermentation. The metabolites of Cluster III were composed of the primary metabolites of carbohydrates and peptides, such as sugars, small peptides, nucleotides, etc. Cluster IV was composed of 169 compounds, and there is no obvious regularity in their concentration distribution, indicating these compounds are greatly disturbed by fermentation or detection conditions.

### 3.3. Metabolite Characterization Based on the Derivatized GC-MS

**Figure 3:**
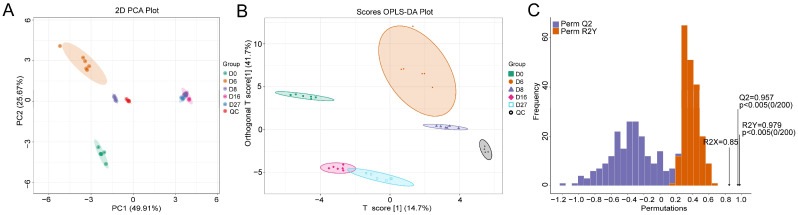
Principle component analysis (PCA) and orthogonal partial least-squares discriminant analysis (OPLS-DA) of the compounds based on the GC-MS detection. (**A**) The PCA model. (**B**) The OPLS-DA score plot of different fermentation samples. (**C**) The 200× permutation tests of different fermentation samples. D0, D6, D8, D16, and D27 represent the fermentation samples collected on 0, 6, 8, 16, and 27 days, respectively.

A total of 83 compounds were annotated by the derivatized GC-MS analysis. All detected samples and QC samples showed distinct clustering in the PCA score plots (Figure 3A). The samples from 16 and 27 days of fermentation overlapped partially. While other samples were assigned to a separate group, indicating that these compounds were changing dynamically during fermentation. When the supervised OPLS-DA model was adopted, all samples from different fermentation times were completely separated (Figure 3B). The OPLS-DA models were cross-validated and underwent 200 permutation tests. An R2X value of 0.85 and an R2Y value of 0.979 were obtained. The value of Q2 was 0.957, which indicated the model had an excellent predictive ability. The *p* values of R2Y and Q2 were all lower than 0.005, implying that the model was statistically valid without overfitting (Figure 3C).

A total 83 compounds were annotated by GC-MS and they comprised organic acids (24, 28.92%), amino acids (21, 25.30%), sugars (9, 10.84%), polyols (8, 9.64%), phosphoric acids (7, 8.43%), fatty acids (5, 6.02%), amines (2, 2.41%) and unclassified compounds (7, 8.43%) according to the KEGG and metabolome database (Table S3). All these compounds were classified into two clusters by HCA (Figure S4). A total of 53 compounds, mainly including 19 amino acids, 16 organic acids (heptanoic acid, aminobutyric acid, lactic acid, succinic acid, hydroxybutanoic acid, etc.), and 5 polyols (mannitol, glycerol, threitol, ribitol, and myo-inositol) were assigned to Cluster II, and their concentrations were obviously increased after 16 and 27 days of fermentation. These compounds were considered to contribute to the flavor generation in NLS. The metabolites of Cluster I were composed of 30 compounds, including 8 sugars, 4 fatty acids, and 11 organic acids, which belong to the primary metabolites. These 30 compounds showed the opposite trends to those in Cluster II, indicating these compounds will be degraded and transformed during fermentation.

### 3.4. Metabolite Characterization Based on HS-SPME-GC-MS

**Figure 4:**
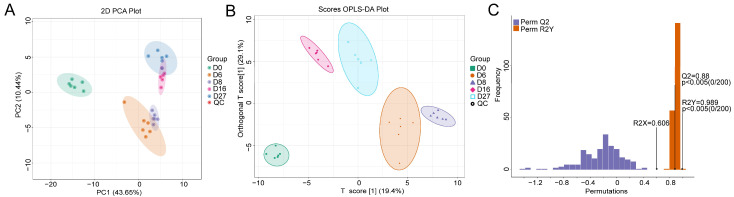
Principle component analysis (PCA) and orthogonal partial least-squares discriminant analysis (OPLS-DA) of the compounds based on the HS-SPME-GC-MS detection. (**A**) The PCA model. (**B**) The OPLS-DA score plot of the different fermentation samples. (**C**) The 200× permutation tests of the different fermentation samples. D0, D6, D8, D16, and D27 represent the fermentation samples collected on 0, 6, 8, 16, and 27 days, respectively.

PCA of the total volatile organic compounds (VOCs) showed a significant difference between QC and the fermented grains samples (Figure 4A). However, the VOCs from the samples of 6 days and 8 days, 16 days and 27 days fermentation were partially overlapped. All VOCs from the different groups were separated well when the supervised OPLS-DA model was used (Figure 4B). The OPLS-DA model was cross-validated and underwent 200 permutation tests. An R2X value of 0.606 and an R2Y value of 0.989 were obtained. The value of Q2 was 0.88, which indicated the model had an excellent predictive ability. The *p* values of R2Y and Q2 were all lower than 0.005, implying that the model was statistically valid without overfitting (Figure 4C).

A total of 181 volatile compounds were identified in the fermented grains by HS-SPME-GC-MS, including esters (75, 41.44%), alcohols (16, 8.84%), aldehydes (10, 5.52%), ketones (6, 3.31%), organic acids (9, 4.97%), alkanes (22, 12.15%), alkenes (20, 11.05%), and unclassified metabolites (23, 12.71%) (Table S4). All compounds were classified into two clusters by HCA (Figure S5). A total of 119 compounds formed Cluster I, and their concentrations were obviously decreased from 6 days of fermentation. Another 62 compounds, including ethyl butyrate, acetic ester, benzyl alcohol, ethanol, and ethyl isovalerate, formed Cluster II, and the dynamic changes in their concentrations showed the opposite trends to that of Cluster I. Based on the analysis, some ethyl esters, alcohols, and aldehydes were strongly correlated with the unique flavor of NLS, indicating these compounds possibly contributed to the flavor generation.

A previous study reported that 12 compounds mainly contributed to the flavor of NLS [9]. In this fermentation batch, 7 of these 12 compounds, including ethyl acetate, ethyl 2-methylbutyrate, ethyl isovalerate, ethyl butyrate, isoamyl acetate, ethyl caprylate and 3-methylbutanol were identified. However, the other five compounds, including ethyl acrylate, ethyl valerate, γ-nonalactone, β-damascenone, and geosmin, were not detected. We postulated that their abundances were below the detectable limits under this condition.

### 3.5. Functional Microorganisms and Volatile Compounds Identified Based on O2PLS-DA Model

**Figure 5:**
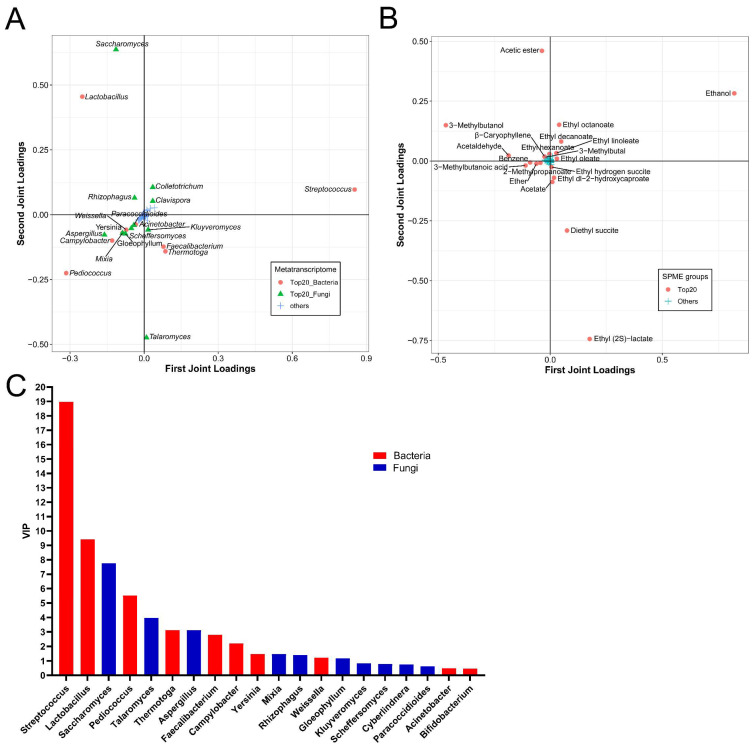
O2PLS-DA model based on the metatranscriptomics and metabolomics at the genus level during NLS fermentation. (**A**) Top 20 genera influencing the flavor compounds based on HS-SPME-GC-MS. (**B**) Top 20 compounds influencing the active genera. (**C**) VIP plot of microbiota at the genus level.

The O2PLS-DA model was used to explore the correlation between the active microorganisms and the volatile flavors during NLS fermentation. A total of 420 active genera were analyzed as X, and 181 volatile flavors were analyzed as Y. The R2X and R2Y values in the model were 0.9823 and 0.9323, respectively, indicating that the O2PLS-DA model was well fitted for analysis and prediction. Then, the top 20 genera with the greatest influence on the formation of flavor compounds were displayed according to the order of the loading scores. They included 9 bacterial genera (*Streptococcus*, *Lactobacillus*, *Pediococcus*, *Thermotoga*, *Faecalibacterium*, *Camylobacter*, *Yersinia*, *Weissella* and *Acinetobacter*) and 11 fungal genera (*Saccharomyces*, *Talaromyces*, *Aspergillus*, *Mixia*, *Rhizophagus*, *Gloeophyllum*, *Scheffersomyces*, *Kluyveromyces*, *Paracoccidioides*, *Clavispora* and *Colletotrichum*) (Figure 5A). Meanwhile, the top 20 volatile metabolites with the great impact on the microbial community were chosen, and they comprised 10 esters including ethyl acetate, ethyl hexanoate, ethyl (2S)-lactate, ethyl octanoate, ethyl hydroxycaproate, ethyl decanoate, ethyl linoleate, ethyl oleate, diethyl succinate and ethyl hydrogen succite, 3 organic acids including acetate, 2-methylpropanoate and 3-methylbutanoic acid, 2 alcohols including ethanol and 3-methylbutanol, 2 aldehydes including acetaldehyde and 3-methylbutal, 1 alkene (β-caryophyllene), and other metabolites of ether and benzene (Figure 5B). Subsequently, the variable sorting was provided according to the importance of the model interpretation by VIP_O2PLS_ profiles for the top 20 genera. The value of VIP varied from 0.45 to 19.11. A total of 14 microbial genera, including 8 bacterial genera and 6 fungal genera with a value of VIP > 1.0, had the most important effects on the volatile flavor generation (Figure 5C). In short, genera of *Streptococcus*, *Lactobacillus*, *Saccharomyces*, *Pediococcus*, *Talaromyces*, *Aspergillus*, *Thermotoga*, *Faecalibacterium*, *Camylobacter*, *Yersinia*, *Mixia*, *Rhizophagus*, *Weissella,* and *Gloeophyllum* were the important contributors to the volatile flavor generation of NLS.

**Figure 6:**
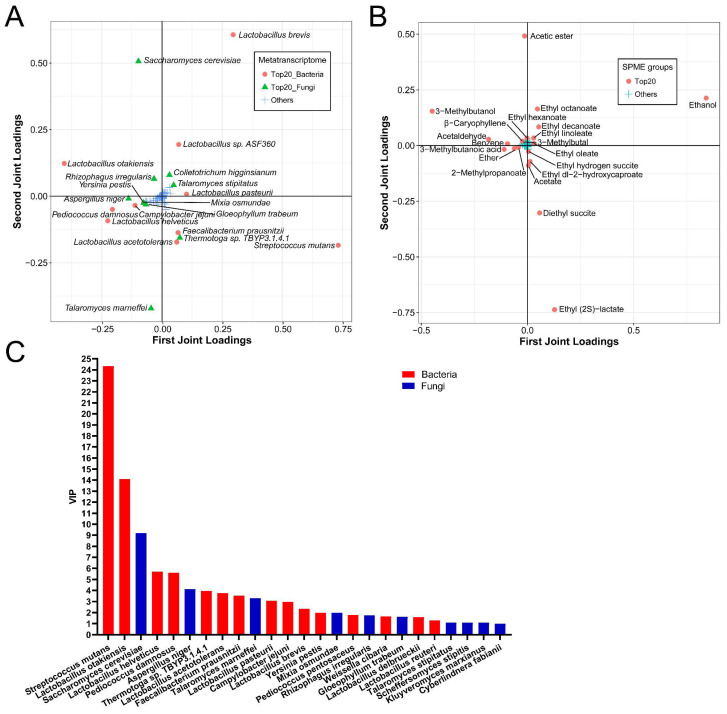
O2PLS-DA model based on the metatranscriptomics and metabolomics at the species level during NLS fermentation. (**A**) Top 20 species influencing the flavor compounds based on HS-SPME-GC-MS. (**B**) Top 20 compounds influencing the active species. (**C**) VIP plot of microbiota at the species level.

Accordingly, the O2PLS-DA model was used to analyze the association between the active species and the volatile compounds. The results indicated that the R2X and R2Y values were 0.9924 and 0.9272, respectively. Then, the top 20 species with the greatest influence on the generation of flavor metabolites were composed of 14 bacterial species, including *S. mutans*, *L. otakiensis*, *L. helveticus*, *P. damnosus*, *Thermotoga* sp. TBYP3.1.4.1, *L. acetotolerans*, *F. prausnitzii*, *L. pasteurii*, *C. jejuni*, *L. brevis*, *Y. pestis*, *P. pentosaceus*, *W. cibaria* and *L. delbrueckii*, and 6 fungal species including *S. cerevisiae*, *A. niger*, *T. marneffei*, *M. osmundae*, *R. irregularis,* and *G. trabeum* (Figure 6A). The top 20 volatile metabolites with the greatest impact on species were displayed (Figure 6B). There were 15 bacterial species and 9 fungal species with a value of VIP > 1.0 among all top 25 species (VIP varied from 0.99–24.33) (Figure 6C). Therefore, except for the top 20 species based on the loading score, four species, including *L. reuteri*, *T. stipitatus*, *S. stipites,* and *K. marxianus,* were the most important species for flavor generation during NLS fermentation.

### 3.6. Correlations between the Active Microorganisms and the Volatile Flavors Based on O2PLS-DA Model

**Figure 7:**
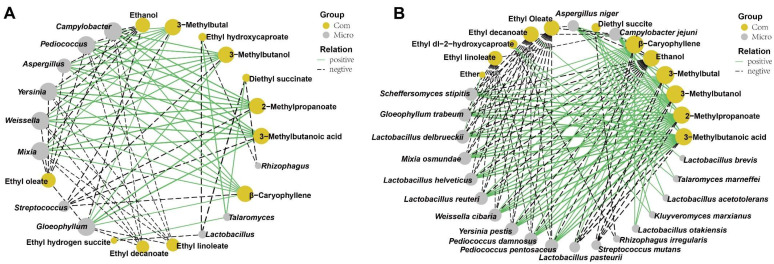
Correlation analysis between microbiota with VIP > 1 and volatile compounds by O2PLS-DA model. (**A**) Significantly correlated network between the important genera with VIP > 1 and the top 20 volatile compounds. (**B**) Significant correlated network between the important species with VIP > 1 and the top 20 volatile compounds. Only strong correlations (|rho| > 0.8, *p* < 0.05)) were displayed. Yellow and grey nodes represent the compounds and t microbiota, respectively.

In order to investigate the correlation of the 14 genera with VIP > 1.0 and the top 20 volatile flavors, Pearson’s correlation analysis was performed, and their correlation was shown in Figure S6A. A total of 73 strong correlations (the absolute value of correlation coefficient ≥ 0.8) were displayed. Among the 14 microorganisms with VIP > 1.0, the bacterial genus of *Streptococcus* showed highly negative correlations with the production of 3-methylbutanoic acid, 3-methylbutal, 2-methylpropanoate, 3-methylbutanol and β-caryophyllene. On the contrary, the fungal genus of *Aspergillus* was positively correlated with the production of 2-methylpropanoate, 3-methylbutal, 3-methylbutanoic acid, 3-methylbutanol, β-caryophyllene, and negatively correlated with the production of ethanol and ethyl oleate. *Lactobacillus* and *Rhizophagus* were negatively correlated with the production of diethyl succinate and ethyl hydroxycaproate. Conversely, the fungal genus of *Talaromyces* was positively correlated with the production of ethyl hydroxycaproate and diethyl succinate. Other bacterial genera, including *Campylobacter*, *Pediococcus*, *Weissella,* and *Yersinia*, and fungal genera, including *Gloeophyllum* and *Mixia,* exhibited positive correlations with the production of 3-methylbutanoic acid, 2-methylpropanoate, 3-methylbutanol, 3-methylbutal and β-caryophyllene, and exhibited negative correlations with the production of ethanol, ethyl oleate, ethyl decanoate and ethyl linoleate (Figure 7A).

The correlation between the 24 species with VIP > 1.0 and the top 20 volatile flavors was investigated based on Pearson’s correlation analysis, and their correlation was shown (Figure S6B). All 130 strong correlations (the absolute value of correlation coefficient ≥ 0.8) were constructed, and 20 species were involved (Figure 7B). Among the 20 species, the bacterial species including *L. reuteri*, *L. helveticus*, *W. cibaria*, *P. pentosaceus*, *P. damnosus*, *Yersinia pestis* and *C. jejuni*, and fungal species including *G. trabeum*, *S. stipites* and *M. osmundae* were positively correlated with the production of 3-methylbutanoic acid, 2-methylpropanoate, 3-methylbutanol, 3-methylbutal and β-caryophyllene, and were negatively correlated with the production of ethanol, ethyl oleate, ethyl decanoate and ethyl linoleate. In addition to exhibiting the above characteristics, *L. delbrueckii* was also positively correlated with ether production. *K. marxianus*, *T. marneffei,* and *L. acetotolerans* had a positive correlation with the production of diethyl succite and ethyl-2-hydroxycaproate. However, *R. irregularis* was negatively correlated with the above two compounds. *L. otakiensis* was positively correlated with the production of 3-methylbutanol, 3-methylbutal and β-caryophyllene. The bacterial species, including *L. pasteurii* and *S. mutans,* were negatively correlated with the production of 3-methylbutanoic acid, 2-methylpropanoate, 3-methylbutanol, 3-methylbutal and β-caryophyllene, and *L. pasteurii* was positively correlated with the ethanol production. The fungal species of *A. niger* displayed the opposite pattern with *L. pasteurii* for the production of 3-methylbutanoic acid, 2-methylpropanoate, 3-methylbutanol, 3-methylbutal, β-caryophyllene and ethanol. Additionally, the bacterial species of *L. brevis* exhibited a positive correlation with ethyl decanoate production.

## 4. Discussion

The multi-omics approach by DNA-based metagenomics coupled with metabolomics was effective in revealing the mechanisms of traditional fermentation [20,21]. However, the microbial community from the metagenomic and metatranscriptomic sequencing varied significantly [22,23]. Metatranscriptomic analysis can help us to find the active functional microbiota [11,24]. Based on the above considerations, integrated metatranscriptomics, and metabolomics analysis were adopted in NLS fermentation, helping to explore the metabolite profiles and the contributed active functional microbiota for flavor generation.

Metabolites from the fermented grains contribute to baijiu flavor generation, and they are the precursors or the final substances [20,25]. In order to comprehensively understand the metabolite profiles of NLS fermentation, three detection approaches, including LC-MS, GC-MS, and HS-SPME-GC-MS, were applied. In LC-MS detection, 395 metabolites were identified, and the number of polar water-soluble metabolites was far more than the 54 compounds reported in other LFB fermentation [8]. Among all the detected compounds, the concentrations of all alcohols and alditols other than 1-hexadecanol were increased with the fermentation. A total of 39 amino acids displayed a similar pattern with alcohols. However, the concentrations of the other 10 amino acids were decreased from days 0 to 28. The concentration of most aldehydes, which contributed to flavor generation, exhibited an increased pattern with fermentation [7,26]. At the early stage of fermentation, carbohydrates and peptides were degraded to small molecular sugars, short peptides, or amino acids, which made the concentration of most sugars decrease until the end of fermentation. However, the contents of some sugars, including glucose, increased to a higher level from 0 to 28 days of fermentation, which might regulate the microbial metabolic diversity in baijiu fermentation [27]. The contents of 58 organic acids, including lactic acid and linoleic acid, were increased with fermentation, which is consistent with the previous reports [28]. Meanwhile, some organic acids undergo esterification to form esters, which could endow mild and pleasant flavors to baijiu [29]. However, the abundances of the other 29 organic acids decreased with the fermentation process, indicating the content changes in organic acids showed a complicated distribution.

For 83 metabolites detected in GC-MS, they contained 2 alcohols, 12 sugars, 31 organic acids, 5 alditols, 19 amino acids, and 14 other compounds according to the compound’s structure. A total of 19 amino acids and 2 alcohols all exhibited an increased pattern with the fermentation, indicating they were rich in the final products and contributed to flavor generation. The number of esters accounted for the highest proportion among all compounds detected in HS-SPME-GC-MS. Ethyl acetate, ethyl lactate, ethyl caprylate, ethyl decanoate, ethyl hexanoate, ethyl oleate, and ethyl laurate were dominant aromatic substances in NLS fermentation, and their contents and proportions endowed the unique flavor of baijiu [30,31,32,33,34,35,36,37,38,39]. Ethyl acetate had a high odor threshold value (32.6 mg/L), and the aromatic contribution was not remarkable [2]. However, ethyl acetate, with the highest production, could give a refreshing fragrance. According to the contribution to aroma, ethyl caprylate was suggested as one of the characteristic esters of LFB [40], and it was the major component among all the compounds. Organic acids had high flavor thresholds and thereby limited influence on the pungent flavors of baijiu [41]. The aromatic compound phenethyl alcohol has a honey rose flavor, and benzaldehyde smells of an almond aroma but is slightly bitter, which likely gives the characteristic aromas of LFB [42]. Meanwhile, β-caryophllene as one healthcare factor from NLS, had a potent scavenging effect against intracellular reactive oxygen species by increasing the activity of intracellular antioxidant enzymes, including catalase, superoxide dismutase, and glutathione peroxidase and the level of non-enzymatic antioxidants such as glutathione [43]. In short, the proportion and combination of all esters, alcohols, organic acids, aldehydes, and other ingredients were all together to form the unique aroma of NLS, which is mellow, sweet, pure, and harmonious.

The correlations between active microbiota at the genus/species level and metabolites proved the genus *Lactobacillus* was the most active bacteria in LFB fermentation. However, different *Lactobacillus* species performed unique functions during NLS fermentation. For example, *L. reuteri*, *L. helveticus,* and *L. delbrueckii* contributed to the production of 3-methylbutanoic acid, 2-methylpropanoate, 3-methylbutanol, 3-methylbutal, β-caryophyllene, ethanol, ethyl oleate, ethyl decanoate and ethyl linoleate. However, *L. pasteurii* had the opposite function for the production of 3-methylbutanoic acid, 2-methylpropanoate, 3-methylbutanol, 3-methylbutal, β-caryophyllene, and ethanol. Additionally, *L. acetotolerans* exhibited special characteristics for the production of diethyl succite and ethyl-2-hydroxycaproate, as the results of the fungal species of *K. marxianus* and *T. marneffei*. These results suggested the succession of *Lactobacillus* with diversified functions is vital for NLS flavor generation. The abundance of *Streptococcus* increased during fermentation, and the contents of 3-methylbutanoic acid, 3-methylbutal, 2-methylpropanoate, 3-methylbutanol, and β-caryophyllene were higher at day 0 and then dropped at the end of fermentation. This might explain why *S. mutants* had a negative correlation with them. Although the diversity and relative abundance of fungi dropped during the NLS fermentation, they also contributed to the flavor generation. For example, *G. trabeum*, *S. stipites,* and *M. osmundae* had positive correlations with the production of 3-methylbutanoic acid, 2-methylpropanoate, 3-methylbutanol, 3-methylbutal, β-caryophyllene, and negative correlations with the production of ethanol, ethyl oleate, ethyl decanoate and ethyl linoleate. *R. irregularis* was negatively correlated with the production of diethyl succite and ethyl-2-hydroxycaproate; however, *K. marxianus* and *T. marneffei* exhibited the opposite function for the above two compounds, implying that the fungi had diversified contributions to NLS flavor.

## 5. Conclusions

Herein, we presented an integrated metatranscriptomic and untargeted metabolomic analysis in NLS fermentation. Not only the metabolite profiles but also the contributed active functional microbiota for key volatile flavors was comprehensively studied based on the O2PLS-DA model. A total of 395, 83, and 181 metabolites were detected in LC-MS, GC-MS, and HS-SPME-GC-MS, respectively. There were 73 and 130 strong correlations between microbes with VIP > 1.0 and the top 20 volatile flavors comprehensively analyzed at the genus and species level, respectively. The correlation analysis revealed that bacterial genera of *Streptococcus*, *Lactobacillus*, *Pediococcus*, *Campylobacter*, *Yersinia,* and *Weissella*, and fungal genera of *Talaromyces*, *Aspergillus*, *Mixia*, *Rhizophagus,* and *Gloeophyllum* were the active functional microbiota for the flavor generation. It was found that different *Lactobacillus* species performed unique functions during NLS fermentation, suggesting the succession of *Lactobacillus* with diversified functions is vital for NLS flavor generation. Our results further improved the understanding of the mechanism in NLS fermentation, which could be useful for improving the LFB quality.

## Data Availability

The metatranscriptomic data presented in this work can be found in the repository NCBI SRA under accession number (PRJNA773513). Metabolomic data used to support the findings of this study can be made available by the corresponding author upon request.

## Supplementary Materials

The following supporting information can be downloaded at: https://www.mdpi.com/article/10.3390/foods12224140/s1, Table S1: Numbers of genera and species in fermented grains based on the metatranscriptomic sequencing; Table S2: 395 polar compounds identified by LC-MS during NLS fermentation; Table S3: 83 compounds identified by derivative GC-MS during NLS fermentation; Table S4: 181 VOCs identified by HS-SPME-GC-MS during NLS fermentation; Figure S1: Fermentation time points and methods of sampling for untargeted metabolomics; Figure S2: Distribution of microorganisms at the superkingdom level; Figure S3: Hierarchical cluster analysis of compounds during NLS fermentation based on the LC-MS detection; Figure S4: Hierarchical cluster analysis of compounds during NLS fermentation based on the GC-MS detection; Figure S5: Hierarchical cluster analysis of compounds during NLS fermentation based on the SPME-GC-MS detection; Figure S6: Correlation analysis between microbiota and volatile compounds by O2PLSDA model.

## References

[1] Wang L. (2022). Research trends in Jiang-flavor baijiu fermentation: From fermentation microecology to environmental ecology. J. Food Sci..

[2] Xu Y., Zhao J., Liu X., Zhang C., Zhao Z., Li X., Sun B. (2022). Flavor mystery of Chinese traditional fermented baijiu: The great contribution of ester compounds. Food Chem..

[3] Gao L., Zhou J., He G. (2022). Effect of microbial interaction on flavor quality in Chinese baijiu fermentation. Front. Nutr..

[4] Ye H., Wang J., Shi J., Du J., Zhou Y., Huang M., Sun B. (2021). Automatic and intelligent technologies of solid-state fermentation process of Baijiu production: Applications, challenges, and prospects. Foods.

[5] Wu Q., Zhu Y., Fang C., Wijffels R., Xu Y. (2021). Can we control microbiota in spontaneous food fermentation?—Chinese liquor as a case example. Trends Food Sci. Technol..

[6] Hong J., Tian W., Zhao D. (2020). Research progress of trace components in sesame-aroma type of baijiu. Food Res. Int..

[7] Liu H., Sun B. (2018). Effect of fermentation processing on the flavor of Baijiu. J. Agric. Food Chem..

[8] Huang X., Fan Y., Lu T., Kang J., Pang X., Han B., Chen J. (2020). Composition and metabolic functions of the microbiome in fermented grain during light-flavor baijiu fermentation. Microorganisms.

[9] Wang Z., Wang Y., Zhu T., Wang J., Huang M., Wei J., Ye H., Wu J., Zhang J., Meng N. (2021). Characterization of the key odorants and their content variation in Niulanshan Baijiu with different storage years using flavor sensory omics analysis. Food Chem..

[10] Ling Y., Li W., Tong T., Li Z., Li Q., Bai Z., Wang G., Chen J., Wang Y. (2020). Assessing the microbial communities in four different Daqus by using PCR-DGGE, PLFA, and biolog analyses. Pol. J. Microbiol..

[11] Pan Y., Wang Y., Hao W., Duan C., Wang S., Wei J., Liu G. (2022). Metatranscriptomics unravel composition, drivers, and functions of the active microorganisms in light-flavor liquor fermentation. Microbiol. Spectr..

[12] Wang J.W., Han P.J., Han D.Y., Zhou S., Li K., He P.Y., Zhen P., Yu H.X., Liang Z.R., Wang X.W. (2021). Genetic diversity and population structure of the amylolytic yeast *Saccharomycopsis fibuligera* associated with Baijiu fermentation in China. J. Microbiol..

[13] Pang X.N., Huang X.N., Chen J.Y., Yu H.X., Wang X.Y., Han B.Z. (2020). Exploring the diversity and role of microbiota during material pretreatment of light-flavor Baijiu. Food Microbiol..

[14] Tang J., Liu Y., Lin B., Zhu H., Jiang W., Yang Q., Chen S. (2021). Effects of ultra-long fermentation time on the microbial community and flavor components of light-flavor Xiaoqu Baijiu based on fermentation tanks. World J. Microbiol. Biotechnol..

[15] Wang Q., Dong K., Wu Y., An F., Luo Z., Huang Q., Wei S. (2022). Exploring the formation mechanism of off-flavor of irradiated yak meat based on metabolomics. Food Chem. X.

[16] Want E.J., Wilson I.D., Gika H., Theodoridis G., Plumb R.S., Shockcor J., Holmes E., Nicholson J.K. (2010). Global metabolic profiling procedures for urine using UPLC-MS. Nat. Protoc..

[17] Jia S., Wang Y., Hu J., Ding Z., Liang Q., Zhang Y., Wang H. (2016). Mineral and metabolic profiles in tea leaves and flowers during flower development. Plant Physiol. Biochem..

[18] Tomita S., Nakamura T., Okada S. (2018). NMR- and GC/MS-based metabolomic characterization of sunki, an unsalted fermented pickle of turnip leaves. Food Chem..

[19] Team R.C. (2022). R: A Language and Environment for Statistical Computing.

[20] Ferrocino I., Bellio A., Giordano M., Macori G., Romano A., Rantsiou K., Decastelli L., Cocolin L. (2018). Shotgun metagenomics and volatilome profile of the microbiota of fermented sausages. Appl. Environ. Microbiol..

[21] Luo L., Song L., Han Y., Zhen P., Han D., Zhao X., Zhou X., Wei Y., Yu H., Han P. (2023). Microbial communities and their correlation with flavor compound formation during the mechanized production of light-flavor Baijiu. Food Res. Int..

[22] Hu X., Wang K., Chen M., Fan J., Han S., Hou J., Chi L., Liu Y., Wei T. (2020). Profiling the composition and metabolic activities of microbial community in fermented grain for the Chinese strong-flavor Baijiu production by using the metatranscriptome, high-throughput 16S rRNA and ITS gene sequencings. Food Res. Int..

[23] Hu Y., Yang Q., Chen D., Fu B., Zhang Y., Zhang Y., Xia X., Peng N., Liang Y., Zhao S. (2021). Study on microbial communities and higher alcohol formations in the fermentation of Chinese Xiaoqu Baijiu produced by traditional and new mechanical technologies. Food Res. Int..

[24] Zhang H., Tan Y., Wei J., Du H., Xu Y. (2022). Fungal interactions strengthen the diversity-functioning relationship of solid-state fermentation systems. mSystems.

[25] Luo A., Yang N., Yang J., Hao J., Zhao J., Shi S., Hu B. (2023). Effects of microbial interspecies relationships and physicochemical parameters on volatile flavors in sorghum-based fermented grains during the fermentation of Shanxi light-flavored liquor. Food Sci. Nutr..

[26] Liu Q., Zhang X., Zheng L., Meng L., Liu G., Yang T., Lu Z., Chai L., Wang S., Shi J. (2023). Machine learning based age-authentication assisted by chemo-kinetics: Case study of strong-flavor Chinese Baijiu. Food Res. Int..

[27] Wang Z., Ji X., Wang S., Wu Q., Xu Y. (2021). Sugar profile regulates the microbial metabolic diversity in Chinese Baijiu fermentation. Int. J. Food Microbiol..

[28] Qian W., Lu Z.M., Chai L.J., Zhang X.J., Li Q., Wang S.T., Shen C.H., Shi J.S., Xu Z.H. (2021). Cooperation within the microbial consortia of fermented grains and pit mud drives organic acid synthesis in strong-flavor Baijiu production. Food Res. Int..

[29] Cong S., Tian K., Zhang X., Lu F., Singh S., Prior B., Wang Z.X. (2019). Synthesis of flavor esters by a novel lipase from *Aspergillus niger* in a soybean-solvent system. 3 Biotech.

[30] Ding X., Wu C., Huang J., Zhou R. (2015). Changes in volatile compounds of Chinese Luzhou-flavor liquor during the fermentation and distillation process. J. Food Sci..

[31] Zha M., Sun B., Wu Y., Yin S., Wang C. (2018). Improving flavor metabolism of *Saccharomyces cerevisiae* by mixed culture with *Wickerhamomyces anomalus* for Chinese Baijiu making. J. Biosci. Bioeng..

[32] Wang X., Wang B., Sun Z., Tan W., Zheng J., Zhu W. (2022). Effects of modernized fermentation on the microbial community succession and ethyl lactate metabolism in Chinese baijiu fermentation. Food Res. Int..

[33] Xu Y., Huang H., Lu H., Wu M., Lin M., Zhang C., Zhao Z., Li W., Zhang C., Li X. (2021). Characterization of an *Aspergillus niger* for efficient fatty acid ethyl ester synthesis in aqueous phase and the molecular mechanism. Front. Microbiol..

[34] Chen Y., Li F., Guo J., Liu G., Guo X., Xiao D. (2014). Enhanced ethyl caproate production of Chinese liquor yeast by overexpressing EHT1 with deleted FAA1. J. Ind. Microbiol. Biotechnol..

[35] Chen Y., Luo W., Gong R., Xue X., Guan X., Song L., Guo X., Xiao D. (2016). Improved ethyl caproate production of Chinese liquor yeast by overexpressing fatty acid synthesis genes with OPI1 deletion. J. Ind. Microbiol. Biotechnol..

[36] Tian T.T., Ruan S.L., Zhao Y.P., Li J.M., Yang C., Cao H. (2022). Multi-objective evaluation of freshly distilled brandy: Characterisation and distribution patterns of key odour-active compounds. Food Chem. X.

[37] Gao W., Fan W., Xu Y. (2014). Characterization of the key odorants in light aroma type chinese liquor by gas chromatography-olfactometry, quantitative measurements, aroma recombination, and omission studies. J. Agric. Food Chem..

[38] Niu Y., Yao Z., Xiao Q., Xiao Z., Ma N., Zhu J. (2017). Characterization of the key aroma compounds in different light aroma type Chinese liquors by GC-olfactometry, GC-FPD, quantitative measurements, and aroma recombination. Food Chem..

[39] Qian Y.L., An Y., Chen S., Qian M.C. (2019). Characterization of Qingke Liquor Aroma from Tibet. J. Agric. Food Chem..

[40] Fan S., Tang K., Xu Y., Chen S. (2020). Characterization of the potent odorants in Tibetan Qingke Jiu by sensory analysis, aroma extract dilution analysis, quantitative analysis and odor activity values. Food Res. Int..

[41] Chen Y., Li P., Liao L., Qin Y., Jiang L., Liu Y. (2021). Characteristic fingerprints and volatile flavor compound variations in Liuyang Douchi during fermentation via HS-GC-IMS and HS-SPME-GC-MS. Food Chem..

[42] Zhao Z., Sugimachi M., Yoshizaki Y., Yin X., Han X.L., Okutsu K., Futagami T., Tamaki H., Takamine K. (2021). Impact of solid-state saccharification on the flavor of rice-flavor baijiu. J. Food Sci..

[43] Dahham S.S., Tabana Y.M., Iqbal M.A., Ahamed M.B., Ezzat M.O., Majid A.S., Majid A.M. (2015). The anticancer, antioxidant and antimicrobial properties of the sesquiterpene β-caryophyllene from the essential oil of *Aquilaria crassna*. Molecules.

