# Supplementary material for: Exploring the Role of Active Functional Microbiota in Flavor Generation by Integrated Metatranscriptomics and Metabolomics during Niulanshan Baijiu Fermentation
Source: Foods. 2023 Nov 15;12(22):4140. doi: 10.3390/foods12224140 (PMC10669994; doi:10.3390/foods12224140)
Supplement: Supplementary file 1 [file foods-12-04140-s001.zip › foods-2662739-supplementary.pdf]

SUPPLEMENTARY MATERIALS

Exploring the role of active functional microbiota in flavor generation by integrated metatranscriptomics and metabolomics during Niulanshan baijiu fermentation

Yuanyuan Pan [1,†], Ying Wang [2,†], Wenjun Hao [2], Sen Zhou [2], Chengbao Duan [1,3], Qiushi Li [1,3], Jinwang Wei [2,*] and Gang Liu [1,3,*]

[1] State Key Laboratory of Mycology, Institute of Microbiology, Chinese Academy of Sciences, Beijing 100101, China; panyy@im.ac.cn (Y.P.); 18813111804@163.com (C.D.); liqiushi192@mails.ucas.ac.cn (Q.L.)

[2] Niulanshan Distillery, Beijing Shunxin Agriculture Company Limited, Beijing 101301, China; wangying.2006h@163.com (Y.W.); hwj1983hwj@126.com (W.H.); ak19861118ka@126.com (S.Z.)

[3] University of Chinese Academy of Sciences, Beijing 100049, China

[*] Correspondence: weijinwang1971@163.com (J.W.); liug@im.ac.cn (G.L.)

[†] These authors contributed equally to this work.

Supplementary Tables

Table S1. Numbers of genera and species in fermented grains based on the metatranscriptomic sequencing.

	Genera					Species				
	D0	D6	D8	D16	D27	D0	D6	D8	D16	D27
> 0.1%	39	25	21	29	26	59	41	37	50	45
> 1%	14	9	10	8	9	19	12	13	11	12

Table S2. 395 polar compounds identified by LC-MS during NLS fermentation.

No	compounds	No	compounds
	**Amino acids**	**201**	Mannitol 1-phosphate
**1**	beta-Alanine	**202**	Arbutin
**2**	2-Aminoacrylic acid	**203**	N-Acetylmuramate
**3**	4-Aminobutyraldehyde	**204**	N-Acetyl-alpha-D-glucosamine 1-phosphate
**4**	Sarcosine	**205**	Ribose 1,5-bisphosphate
**5**	1-Aminocyclopropanecarboxylic acid	**206**	Sucrose
**6**	L-Serine	**207**	Cellobiose
**7**	gamma-Aminobutyric acid	**208**	D-Maltose
**8**	2-Phenylethanol	**209**	Trehalose
**9**	p-Cresol	**210**	Digalacturonate
**10**	Pyrrole-2-carboxylic acid	**211**	Trehalose 6-phosphate
**11**	Imidazole-4-acetaldehyde	**212**	Stachyose
**12**	Histamine	**213**	R-Methylmalonyl-CoA
**13**	Creatinine		**Cofactors and vitamins**
**14**	(S)-1-Pyrroline-5-carboxylate	**214**	Niacinamide
**15**	1-Pyrroline-2-carboxylic acid	**215**	Nicotinic acid
**16**	L-Proline	**216**	Gamma-delta-Dioxovaleric acid
**17**	5-Aminopentanoic acid	**217**	p-Aminobenzoic acid
**18**	Guanidoacetic acid	**218**	6-Hydroxynicotinic acid
**19**	Methylmalonic acid	**219**	5-(2-Hydroxyethyl)-4-methylthiazole
**20**	Indole	**220**	Methylitaconate
**21**	Betaine	**221**	Maleic acid homopolymer
**22**	L-Valine	**222**	2-Dehydropantoate
**23**	Phenylacetaldehyde	**223**	Pimelic acid
**24**	L-Allothreonine	**224**	Quinolinic acid
**25**	L-Threonine	**225**	Homogentisic acid
**26**	Phenylethylamine	**226**	Pyridoxamine
**27**	Picolinic acid	**227**	Pyridoxine
**28**	Ciliatine	**228**	Ascorbate
**29**	(S)-2,3,4,5-tetrahydropyridine-2-carboxylate	**229**	L-Gulonolactone
**30**	Creatine	**230**	4-Pyridoxic acid
**31**	Pyrrolidonecarboxylic acid	**231**	8-Amino-7-oxononanoate
**32**	Pyroglutamic acid	**232**	Diketogulonic acid
**33**	L-Pipecolic acid	**233**	Galactaric acid
**34**	L-Glutamic gamma-semialdehyde	**234**	Dethiobiotin
**35**	5-Amino-2-oxopentanoic acid	**235**	Pantothenic acid
**36**	Glutaric acid	**236**	Pyrimidodiazepine
**37**	Ureidopropionic acid	**237**	4-Amino-4-deoxychorismate
**38**	L-Leucine	**238**	Porphobilinogen

**39**	cis-4-Hydroxy-D-proline	**239**	Biotin
**40**	3-Methylindole	**240**	Lumichrome
**41**	N-Carbamoylputrescine	**241**	Nicotinate D-ribonucleoside
**42**	L-Isoleucine	**242**	4-(beta-D-Glucosyloxy)benzoate
**43**	L-Asparagine	**243**	D-4-Phosphopantothenate
**44**	Ornithine	**244**	2,5-Diamino-6-(5-phospho-D-ribosylamino)pyrimidin-4(3H)-one
**45**	L-Aspartic acid	**245**	Riboflavin
**46**	Phenylacetic acid	**246**	S-Adenosylmethionine
**47**	3-Hydroxyanthranilic acid	**247**	Folic acid
**48**	4-Hydroxyphenylacetaldehyde	**248**	13(1)-Oxo-magnesium-protoporphyrin IX 13-monomethyl ester
**49**	2-Phenylacetamide		**Energy**
**50**	2-Aminobenzoic acid	**249**	Dimethyl sulfone
**51**	Tyramine	**250**	Hydrogen phosphate
**52**	Gentisate aldehyde	**251**	3-Methylthiopropionic acid
**53**	Urocanic acid	**252**	N-methyl-L-glutamic Acid
**54**	Ectoine	**253**	Pyrophosphate
**55**	Spermidine	**254**	Fructose 1,6-bisphosphate
**56**	2-Keto-glutaramic acid		**Lipid**
**57**	4-Oxoglutaramate	**255**	Acetoacetic acid
**58**	2-Keto-6-aminocaproate	**256**	Butyric acid
**59**	4-Guanidinobutanoic acid	**257**	Glycerol
**60**	(2R,5S)-2,5-Diaminohexanoate	**258**	Malonate
**61**	L-Glutamine	**259**	2-Hydroxybutyric acid
**62**	L-Lysine	**260**	Maltol
**63**	(3S)-3,6-Diaminohexanoate	**261**	O-Phosphoethanolamine
**64**	trans-Cinnamate	**262**	Caprylic acid
**65**	L-Glutamic acid	**263**	Acetylcholine
**66**	O-Acetylserine	**264**	L-2-Hydroxyglutaric acid
**67**	1-Phenyl-1,2-propanedione	**265**	2-Oxo-4-methylthiobutanoic acid
**68**	L-Methionine	**266**	(+)-(S)-Carvone
**69**	3,4-Dihydroxyphenylglycol	**267**	Perillyl aldehyde
**70**	3,4-Dihydroxyphenylacetaldehyde	**268**	rac-Glycerol 3-phosphoate
**71**	Ortho-Hydroxyphenylacetic acid	**269**	Limonene-1,2-diol
**72**	Vanillin	**270**	Phosphorylcholine
**73**	L-Histidine	**271**	Dodecanoic acid
**74**	Gentisic acid	**272**	(+)-7-Isojasmonic acid
**75**	3-Indoleacetonitrile	**273**	Jasmonic acid
**76**	Isopropylmaleate	**274**	Capsidiol
**77**	5-Acetamidovalerate	**275**	2-trans,6-trans-Farnesal
**78**	Indoleacetaldehyde	**276**	Methyl jasmonate
**79**	Oxoadipic acid	**277**	Traumatic Acid
**80**	Tryptophanol	**278**	Myristic acid

**81**	D-4-Hydroxy-2-oxoglutarate	**279**	1-Hexadecanol
**82**	Phenylpyruvic acid	**280**	Xanthoxin
**83**	m-Coumaric acid	**281**	Abscisic alcohol
**84**	Formylanthranilic acid	**282**	Palmitic acid
**85**	L-Methionine S-oxide	**283**	Glycerophosphocholine
**86**	D-Phenyllactic acid	**284**	(S)-Abscisic acid
**87**	L-Phenylalanine	**285**	Xanthoxic acid
**88**	3,4-Dihydroxybenzeneacetic acid	**286**	Stearic acid
**89**	1-Methylhistidine	**287**	Stearidonic acid
**90**	Norepinephrine	**288**	Alpha-Linolenic acid
**91**	Tetrahydrodipicolinate	**289**	Linoleic acid
**92**	3-Dehydroshikimate	**290**	Oleic acid
**93**	N-Acetyl-L-glutamate 5-semialdehyde	**291**	Sphingosine
**94**	1H-Indole-3-acetamide	**292**	9(S)-HPOT
**95**	N-Acetylornithine	**293**	12-OPDA
**96**	L-Arginine	**294**	13(S)-HpOTrE
**97**	N-Formyl-L-methionine	**295**	3-Dehydrosphinganine
**98**	Citrulline	**296**	Sphinganine
**99**	Hippuric acid	**297**	19(S)-HETE
**100**	4-Hydroxyphenylpyruvic acid	**298**	Arachidonic acid
**101**	L-Homophenylalanine	**299**	2,3-Dinor-8-iso prostaglandin F2alpha
**102**	3,4-Dihydroxyhydrocinnamic acid	**300**	Phytosphingosine
**103**	(R)-3-(4-Hydroxyphenyl)lactate	**301**	5-KETE
**104**	Beta-Tyrosine	**302**	12-Keto-tetrahydro-leukotriene B4
**105**	L-Tyrosine	**303**	12-KETE
**106**	Homovanillic acid	**304**	Prostaglandin E2
**107**	Se-Methylselenocysteine	**305**	Prostaglandin-c2
**108**	Epinephrine	**306**	8,9-DiHETrE
**109**	3,4-Dihydroxymandelic acid	**307**	6-Ketoprostaglandin E1
**110**	Normetanephrine	**308**	Prostaglandin G2
**111**	Phosphohydroxypyruvic acid	**309**	Lithocholic acid
**112**	Phosphoserine	**310**	Cholesterol
**113**	Vanylglycol	**311**	Deoxycholic acid
**114**	Kynurenic acid	**312**	3-Keto-4-methylzymosterol
**115**	2-Keto-6-acetamidocaproate	**313**	Lanosterin
**116**	N6-Acetyl-L-lysine	**314**	beta-Carotene
**117**	N-Acetylglutamic acid		**Nucleotide**
**118**	Diaminopimelic acid	**315**	Cytosine
**119**	5-Hydroxyindoleacetic acid	**316**	Uracil
**120**	Selenomethionine	**317**	Dihydrouracil
**121**	L-Dopa	**318**	Thymine
**122**	gamma-Glutamyl-beta-aminopropiononitrile	**319**	(R)-5,6-Dihydrothymine
**123**	4-Fumarylacetoacetate	**320**	Oxalureate

**124**	Xanthurenic acid	**321**	Adenine
**125**	L-Tryptophan	**322**	Hypoxanthine
**126**	4-(2-Aminophenyl)-2,4-dioxobutanoic acid	**323**	5-Methylbarbiturate
**127**	N-Acetyl-L-phenylalanine	**324**	Guanine
**128**	L-Kynurenine	**325**	Xanthine
**129**	N-Acetyldemethylphosphinothricin	**326**	4,5-Dihydroorotic acid
**130**	2-Hydroxy-6-ketononatrienedioate	**327**	Uric acid
**131**	beta-Alanyl-L-lysine	**328**	Deoxyuridine
**132**	gamma-Glutamylalanine	**329**	Cytidine
**133**	D-Lysopine	**330**	Thymidine
**134**	L-Cystine	**331**	Pseudouridine
**135**	5-(Methylthio)-2,3-dioxopentyl phosphate	**332**	Uridine
**136**	beta-Alanyl-L-arginine	**333**	2-Deoxyadenosine
**137**	D-Octopine	**334**	Inosine
**138**	Linatine	**335**	Adenosine
**139**	5-Methylthioribulose 1-phosphate	**336**	Deoxyguanosine
**140**	Subaphylline	**337**	Guanosine
**141**	(2R,3R)-3-Methylglutamyl-5-semialdehyde-N6-lysine	**338**	Xanthosine
**142**	N2-Succinyl-L-arginine	**339**	dGMP
**143**	4-Hydroxycinnamoylagmatine	**340**	3-AMP
**144**	Saccharopine	**341**	AMP
**145**	N-Succinyl-2-amino-6-ketopimelate		**Peptide**
**146**	Glutathione	**342**	Carnosine
**147**	S-Adenosylhomocysteine		**Unknown**
	**Carbohydrate**	**343**	2-Methyl-3-oxopropanoic acid
**148**	3-Butynoate	**344**	5-Aminopentanal
**149**	Pyruvic acid	**345**	D-1-Piperideine-2-carboxylic acid
**150**	Diacetyl	**346**	3-Amino-4-hydroxybenzoate
**151**	L-Lactic acid	**347**	L-4-Hydroxyphenylglycine
**152**	Maleic acid	**348**	Choline sulfate
**153**	Succinic acid semialdehyde	**349**	D-Arabinose 5-phosphate
**154**	2-Ketobutyric acid	**350**	3-Deoxy-D-manno-octulosonate
**155**	(R)-3-Hydroxybutyric acid		**Xenobiotics**
**156**	Glyceric acid	**351**	Cyclopropanecarboxylate
**157**	Fumaric acid	**352**	m-Cresol
**158**	Succinic acid	**353**	Phenol
**159**	Deoxyribose	**354**	Aniline
**160**	Itaconic acid	**355**	Sulfate
**161**	Mesaconate	**356**	Benzaldehyde
**162**	Citraconic acid	**357**	Catechol
**163**	L-Malic acid	**358**	Hydroquinone
**164**	Acetylphosphate	**359**	2-Aminophenol

**165**	L-Fucose	**360**	4-Aminophenol
**166**	Oxoglutaric acid	**361**	Epsilon-caprolactam
**167**	D-Xylonate	**362**	epsilon-Caprolactone
**168**	D-Ribose	**363**	3-Hydroxybenzoic acid
**169**	D-Xylose	**364**	4-Methylbenzaldehyde
**170**	D-Lyxose	**365**	Benzoate
**171**	Tartaric acid	**366**	3-Hydroxybenzyl alcohol glucoside
**172**	L-Arabinose	**367**	4-Methylcatechol
**173**	L-Arabitol	**368**	2-Hydroxybenzaldehyde
**174**	D-Xylitol	**369**	3-Hydroxybenzaldehyde
**175**	Ribitol	**370**	Erythritol
**176**	2-Hydroxy-3-oxoadipate	**371**	4-Aminocatechol
**177**	Alpha-D-Glucose	**372**	Aminohydroquinone
**178**	L-Rhamnono-1,4-lactone	**373**	1,2,3-Trihydroxybenzene
**179**	D-Fructose	**374**	1,3,5-Trihydroxybenzene
**180**	L-Rhamnofuranose	**375**	Adipate semialdehyde
**181**	Gluconolactone	**376**	Aminocaproic acid
**182**	Galactonic acid	**377**	m-toluate
**183**	Glucosamine	**378**	(R)-mandelic Acid
**184**	Allose	**380**	4-Hydroxybenzoic acid
**185**	D-Mannose	**381**	Salicylic acid
**186**	L-Sorbose	**382**	2-Methylbenzoic acid
**187**	myo-Inositol	**383**	Cuminaldehyde
**188**	Galactitol	**384**	3-Hydroxyphenylacetic acid
**189**	Sorbitol	**385**	2-Pyrocatechuic acid
**190**	Mannitol	**386**	Protocatechuic acid
**191**	Citric acid	**387**	3-Oxoadipic acid
**192**	Isocitric acid	**388**	cis-1,2-Dihydronaphthalene-1,2-diol
**193**	D-Glucuronic Acid	**389**	4-Hydroxycinnamic acid
**194**	Gluconic acid	**390**	Gallic acid
**195**	N-Acetyl-D-glucosamine	**391**	(S)-4-Hydroxymandelate
**196**	Inositol phosphate	**392**	1-Hydroxy-2-naphthoate
**197**	D-Mannose 1-phosphate	**393**	2,4-Dinitrotoluene
**198**	Glucose 6-phosphate	**394**	Syringic acid
**199**	Fructose 6-phosphate	**395**	5-Carboxyvanillic acid
**200**	Beta-D-Glucose 6-phosphate		

Table S3. 83 compounds identified by derivative GC-MS during NLS fermentation.

No	compounds	No	compounds
	**amine**	**43**	Quinic acid
**1**	Ethanolamine	**44**	Ribonic acid
**2**	Histamine	**45**	Shikimic acid
	**Amino acids**	**46**	Succinic acid
**3**	2-Aminobutyric acid	**47**	Threonic acid
**4**	4-Aminobutyric acid	**48**	Trans-Ferulic acid
**5**	Alanine	**49**	2,4-Dihydroxybutanoic acid
**6**	Asparagine	**50**	4-Hydroxy-3-Methoxybenzoic acid
**7**	Aspartic acid	**51**	4-Hydroxybutanoic acid
**8**	Beta-Alanine	**52**	Dl-Beta-Hydroxybutyric acid
**9**	Glutamic acid		**Others**
**10**	Glutamine	**53**	Adenosine
**11**	Glycine	**54**	Threonic acid-1,4-Lactone
**12**	Isoleucine	**55**	Uracil
**13**	Leucine	**56**	Urea
**14**	Lysine	**57**	Uridine
**15**	Methionine	**58**	Hypoxanthine
**16**	Phenylalanine	**59**	Thymine
**17**	Proline		**Phosphoric acid**
**18**	Pyroglutamic acid	**60**	Glucose-6-Phosphate
**19**	Serine	**61**	Glycerol-3-Phosphate
**20**	Threonine	**62**	Monomethylphosphate
**21**	Tyrosine	**63**	Myo-Inositol-1-Phosphate
**22**	Valine	**64**	Phosphoric acid
**23**	Ornithine	**65**	Fructose-6-Phosphate
	**Fatty acid**	**66**	Sorbitol-6-Phosphate
**24**	Heptanoic acid		**Polyol**
**25**	Hexadecanoic acid	**67**	Erythritol
**26**	Octadecanoic acid	**68**	Glycerol
**27**	9-(Z)-Octadecenoic acid	**69**	Mannitol
**28**	9,12-(Z,Z)-Octadecadienoic acid	**70**	Myo-Inositol
	**Organic acid**	**71**	Sorbitol
**29**	2-Hydroxyglutaric acid	**72**	1-Monohexadecanoylglycerol
**30**	2-Keto-L-Gluconic acid	**73**	Ribitol
**31**	Citric acid	**74**	Threitol
**32**	Erythronic acid		**Sugar**
**33**	Fumaric acid	**75**	Fructose
**34**	Galactaric acid	**76**	Galactose
**35**	Glyceric acid	**77**	Glucose
**36**	Glycolic acid	**78**	Maltose
**37**	Lactic acid	**79**	Ribose

**38**	Malic acid	**80**	Sucrose
**39**	Nicotinic acid	**81**	Trehalose
**40**	Oxalic acid	**82**	Fucose
**41**	Parabanic acid	**83**	Gentiobiose
**42**	Pyruvic acid		

Table S4. 181 VOCs identified by HS-SPME-GC-MS during NLS fermentation.

No	compounds	No	compounds
	**Esters**	93	1-Octen-3-ol
1	Nonoic acid, 2,4,6-trimethyl-, methyl ester, (2R,4S,6R)-(-)-	94	1,1,3,3,5,5,7,7-Octamethyl-7-(2-methylpropoxy)tetrasiloxan-1-ol
2	Ethyl formate	95	2-Furanmethanol
3	Arsenous acid tris(trimethylsilyl) ester	96	3-(Methylthio)-1-propanol
4	Acetic ester	97	Benzyl alcohol
5	2-Methylpropyl acetate	98	Phenylethyl alcohol
6	Ethyl butyrate	99	4aH-Cycloprop[e]azulen-4a-ol, decahydro-1,1,4,7-tetramethyl-
7	Ethyl 2-methylbutyrate	100	(-)-Epiglobulol
8	Ethyl isovalerate	101	noethylene glycol
9	Isoamyl acetate	102	Prenol
10	Ethyl hexanoate		**Ketones**
11	Sulfurous acid, butyl nonyl ester	103	2,3-Butanedione
12	Pentyl formate	104	2,3-Pentanedione
13	Ethyl 5-methylhexanoate	105	4-Heptanone
14	Isoamyl isovalerate	106	2-Heptanone
15	Ethyl heptanoate	107	3-Octanone
16	Docosanoic acid, ethyl ester	108	2-Nonone
17	Ethyl lactate		**Acids**
18	ethyl (2S)-lactate	109	Acetate
19	4-Heptenoic acid, ethyl ester, (E)-	110	Propanoic acid
20	Methyl caprylate	111	Orsellite
21	Ethyl octanoate	112	3-Methylbutanoic acid
22	3-Methylbutyl hexanoate	113	Hexanoic acid
23	Ethyl 4Z-octenoate	114	Octanoic acid
24	Ethyl nonoate	115	Octanoic acid, silver(1+) salt
25	Ethyl dl-2-hydroxycaproate	116	Lactic acid
26	n-Caprylic acid isobutyl ester	117	Linoleate
27	Octyl formate		**Alkene**
28	Ethyl 3-(methylthio)propanoate	118	cis-2-Octene
29	2-Methylpropanoate	119	trans-3-Octene
30	Methoxyacetic acid, 3-methylbutyl ester	120	Styrene
31	Phosphonessigsaeure-tri-(trimethylsilylester)	121	prehnitene
32	gamma-Butyrolactone	122	Cyperene
33	4-Decenoic acid, methyl ester	123	1-Epi-bicyclosesquiphellandrene
34	Nonoic acid, 2,6-dimethyl-, methyl ester	124	Aristolene
35	Ethyl decanoate	125	Berkheyaradulene
36	Ethyl 4-decenoate	126	(-)-alpha-Gurjunene
37	Diethyl succite	127	beta-Caryophyllene

**38**	ethyl 9-decenoate	**128**	2-Isopropenyl-4a,8-dimethyl-1,2,3,4,4a,5,6,8a-octahydrophthalene
**39**	Succinic acid, ethyl 2-methylphenyl ester	**129**	Cedrene-V6
**40**	Ethyl phenylacetate	**130**	valencene
**41**	Succinic acid, ethyl 3-heptyl ester	**131**	(1R,4aS,8aS)-7-methyl-4-methylidene-1-propan-2-yl-2,3,4a,5,6,8a-hexahydro-1H-phthalene
**42**	4-Ethylbenzoic acid, dodec-9-ynyl ester	**132**	alpha-muurolene
**43**	Phenethyl acetate	**133**	.gamma.-Elemene
**44**	Methyl 3-phenylpropanoate	**134**	delta-Cadinene
**45**	Lauric Acid ethyl ester	**135**	Caryophyllene epoxide
**46**	Ethyl 3-phenylpropanoate	**136**	Longifolene
**47**	1-O-ethyl 4-O-(3-methylbutyl) butanedioate	**137**	gamma-gurjunene
**48**	methyl 2-methyltetradecanoate		**Alkanes**
**49**	Tetradecanoic acid, ethyl ester	**138**	2,4-dimethylheptane
**50**	Ethyl 13-methyl-tetradecanoate	**139**	Octamethyltrisiloxane
**51**	Octadecanoic acid, 9-methyl-, ethyl ester	**140**	Methylene chloride
**52**	Ethyl cinmate	**141**	2,2,4,6,6-pentamethylheptane
**53**	Pentadecanoic acid, ethyl ester	**142**	N-Decane
**54**	(Z)-Ethyl pentadec-9-enoate	**143**	trisiloxane, 1,1,1,5,5,5-hexamethyl-3-[(trimethylsilyl)oxy]-
**55**	Palmitic Acid ethyl ester	**144**	Decane, 3,7-dimethyl-
**56**	Methyl hexadec-9-enoate	**145**	Undecane
**57**	Ethyl 9-hexadecenoate	**146**	Decamethyl-Tetrasiloxane
**58**	Oxalic acid, pentyl 2-phenylethyl ester	**147**	3,8-Dimethyldecane
**59**	methyl 17-methyloctadecanoate	**148**	3-methyl-Undecane
**60**	Dihydroactinidiolide	**149**	Decamethylcyclopentasiloxane
**61**	Heptadecanoic acid, ethyl ester	**150**	Dodecane, 1-iodo-
**62**	(Z)-Ethyl heptadec-9-enoate	**151**	Dodecane
**63**	Ethyl hydrogen succite	**152**	heptacosane
**64**	Stearic Acid ethyl ester	**153**	Eicosane, 1-iodo-
**65**	Oleic Acid ethyl ester	**154**	Tridecane
**66**	cis-10-Pentadecenoic acid, propyl ester	**155**	Heptadecane
**67**	octadeca-9,12-dienoic acid ethyl ester	**156**	tetradecane
**68**	Phthalic acid, isobutyl 4-methylpent-2-yl ester	**157**	dodecamethylpentasiloxane
**69**	Linolenic Acid ethyl ester	**158**	3-(1,3-Dihydroxyisopropyl)-1,5,8,11-tetraoxacyclotridecane
**70**	Ethyl 9.cis.,11.trans.-octadecadienoate	**159**	1,4,7,10,13,16-Hexaoxanodecane, 18-(2-propenyl)-
**71**	Linoleic Acid ethyl ester		**Others**
**72**	7,10-Hexadecadienoic acid, methyl ester	**160**	Ether
**73**	n-Propyl 9,12-octadecadienoate	**161**	Benzene

**74**	Dibutyl phthalate	**162**	Chloroform
**75**	Ethyl 3-(4-hydroxy-3-methoxyphenyl)propionate	**163**	Toluene
	**Aldehydes**	**164**	Ethylbenzene
**76**	Acetaldehyde	**165**	m-Xylene
**77**	isobutyraldehyde	**166**	Oxan-4-one, 2-trifluoromethyl-2-hydroxy-5,5-dimethyl-
**78**	2-Methylbutal	**167**	2-Pentylfuran
**79**	3-Methylbutal	**168**	Acetoin
**80**	hexal	**169**	3,4-Dihydrocoumarin, 4,4-dimethyl-6-ethyl-
**81**	nol	**170**	2-Methoxyphenol
**82**	(E)-Hexadec-2-el	**171**	2-Methoxy-4-methylphenol
**83**	Benzaldehyde	**172**	Phenol
**84**	Phenylacetaldehyde	**173**	4-Ethyl-2-methoxyphenol
**85**	Benzaldehyde, 3,5-dimethyl-	**174**	4-Ethylphenol
	**Alcohols**	**175**	Phenthrene, tetradecahydro-
**86**	Ethanol	**176**	15-crown-5
**87**	Propane-1-ol	**177**	2-[2-[2-[2-[2-[2-[2-[2-[2-(2-Methoxyethoxy)ethoxy]ethoxy]ethoxy]ethoxy]ethoxy]ethoxy]ethoxy]etho
**88**	Isobutyl alcohol	**178**	2-[2-[2-[2-[2-[2-[2-[2-[2-[2-(2-Hydroxyethoxy)ethoxy]ethoxy]ethoxy]ethoxy]ethoxy]ethoxy]ethoxy]e
**89**	1-Butanol	**179**	18-crown-6
**90**	3-Methylbutanol	**180**	Oxime-, methoxy-phenyl-_
**91**	isopentenyl alcohol	**181**	4-Vinylguaiacol
**92**	1-Hexanol		

Time	A	B	C	D	E	F	G
D0	√	√	√				FS
D6				√	√	√	FS
D8	√	√	√				FS
D16				√	√	√	FS
D27				√	√	√	FS

√: Two fermented grain samples were collected from each earthen jar

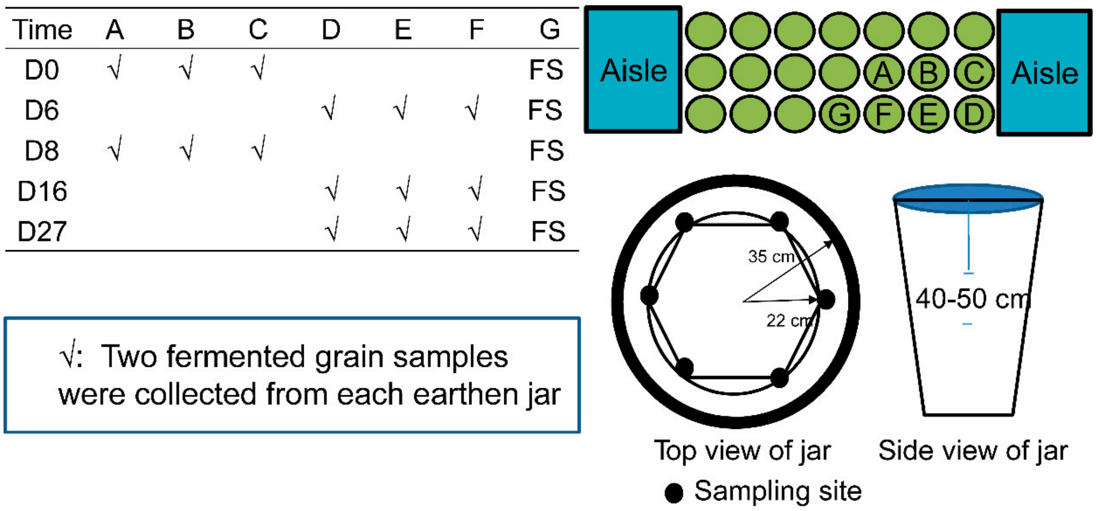

Figure S1. Fermentation time points and methods of sampling for untargeted metabolomics. D0 represents the samples isolated from the fermentation start time. Samples from A, B and C earthen jars were named as D0A, D0B and D0C, respectively. D6 represents the samples isolated from the 6 day of fermentation. Samples from D, E and F earthen jars were named as D6A, D6B and D6C, respectively. The following samples were named in the same way. Both fermented grain samples were collected from each earthen jar at the same time. Samples located between 40 and 50 cm in the earthen jars were collected and mixed well immediately for use, and then supply the same amount of feedstock from earthen jar G to the sampling spaces. FS, Feedstock supply earthen jar; cm, centimeter.

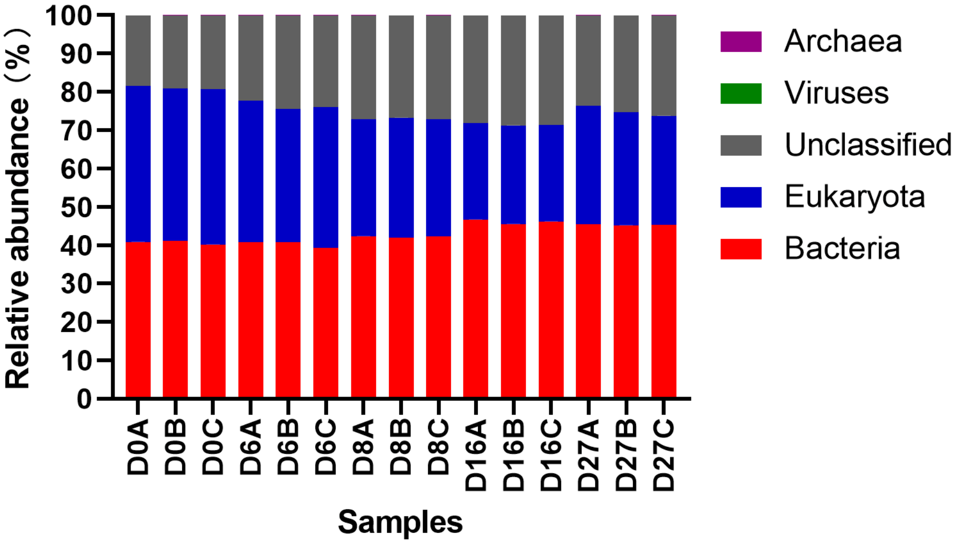

Figure S2. Distribution of microorganisms at the superkingdom level.

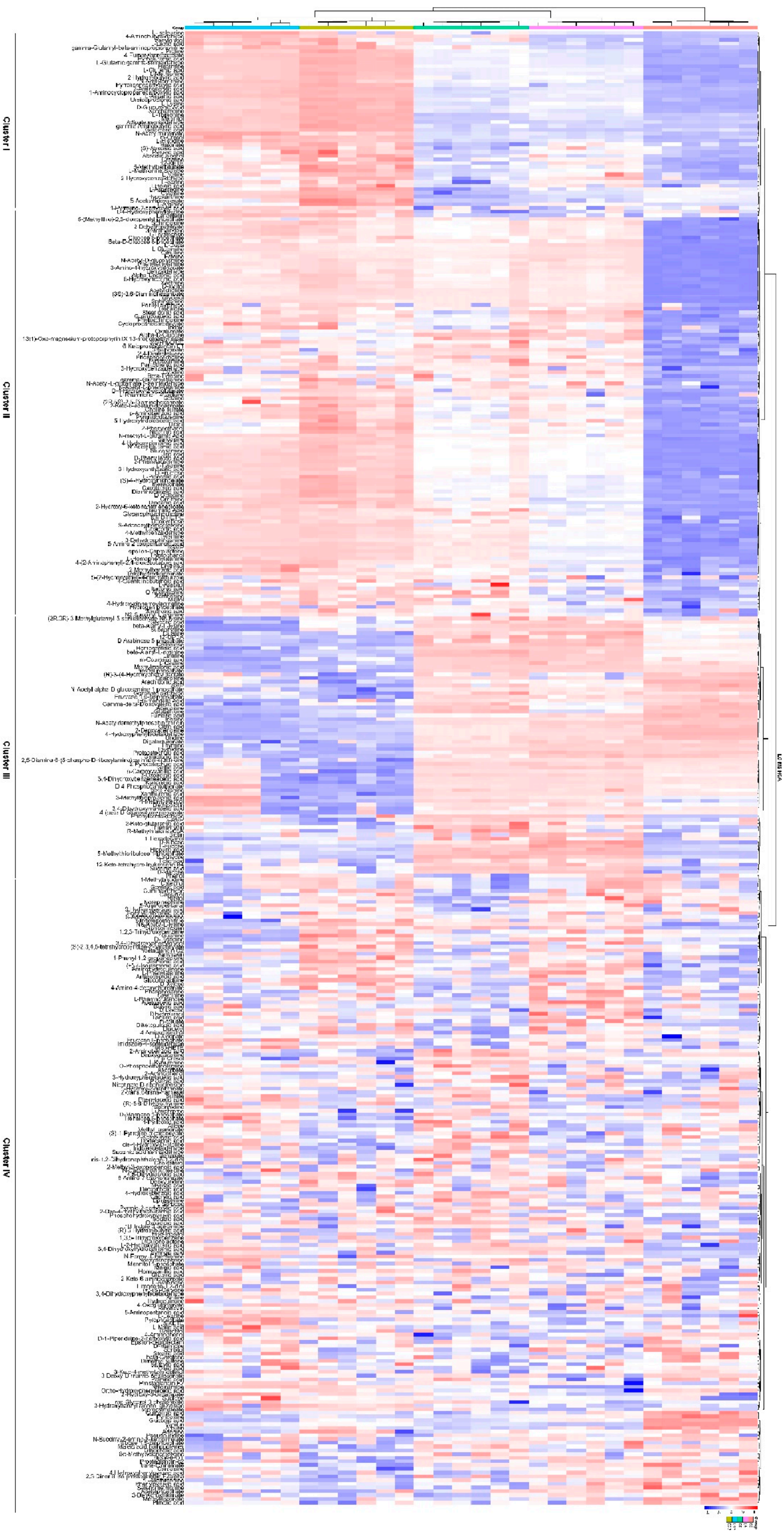

Cluster I
Cluster II
Cluster III
Cluster IV

Figure S3. Hierarchical cluster analysis of compounds during NLS fermentation based on the LC-MS detection. Rows represent the compounds and columns from different batches. Color key indicates the relative abundance of different compounds, green indicates the lowest and red indicates the highest. D0, D6, D8, D16 and D27 represent the fermentation sample of 0, 6, 8, 16 and 27 days, respectively.

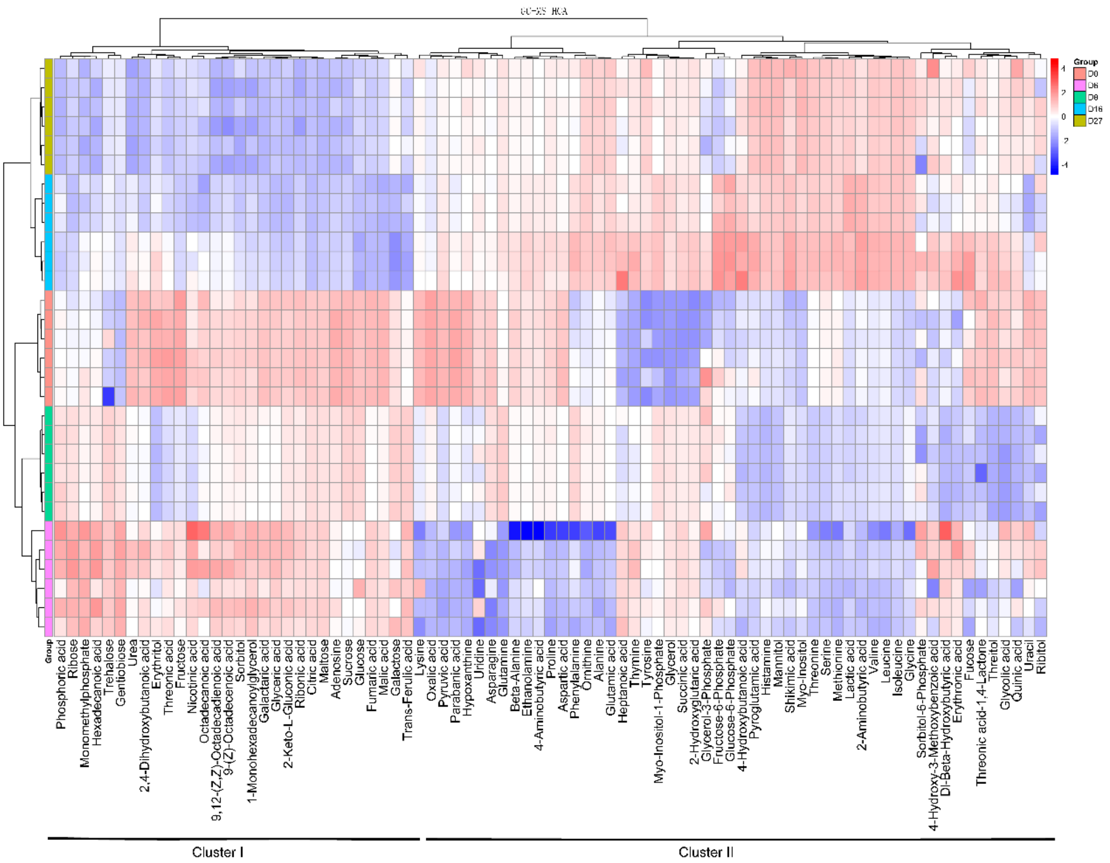

Figure S4. Hierarchical cluster analysis of compounds during NLS fermentation based on the GC-MS detection. Rows represent the compounds and columns represent different batches. Color key indicates the relative abundance of differential compounds, green indicates the lowest and red indicates the highest. D0, D6, D8, D16 and D27 represent the fermentation sample collected on 0, 6, 8, 16 and 27 day, respectively.

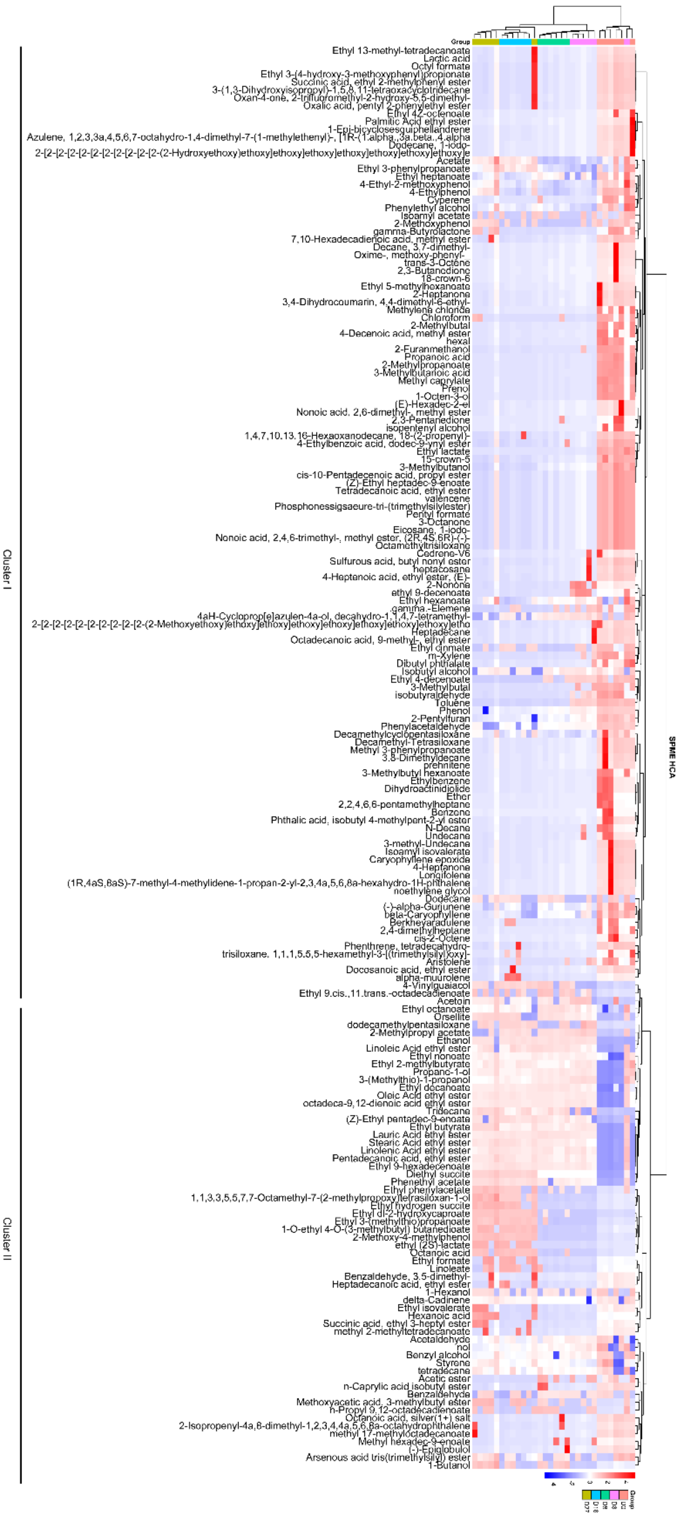

Figure S5. Hierarchical cluster analysis of compounds during NLS fermentation based on the SPME-GC-MS detection. Rows represent the compounds and columns represent different batches. Color key indicates the relative abundance of differential compounds, green indicates the lowest and red indicates the highest. D0, D6, D8, D16 and D27 represent the fermentation sample collected on 0, 6, 8, 16 and 27 day, respectively.

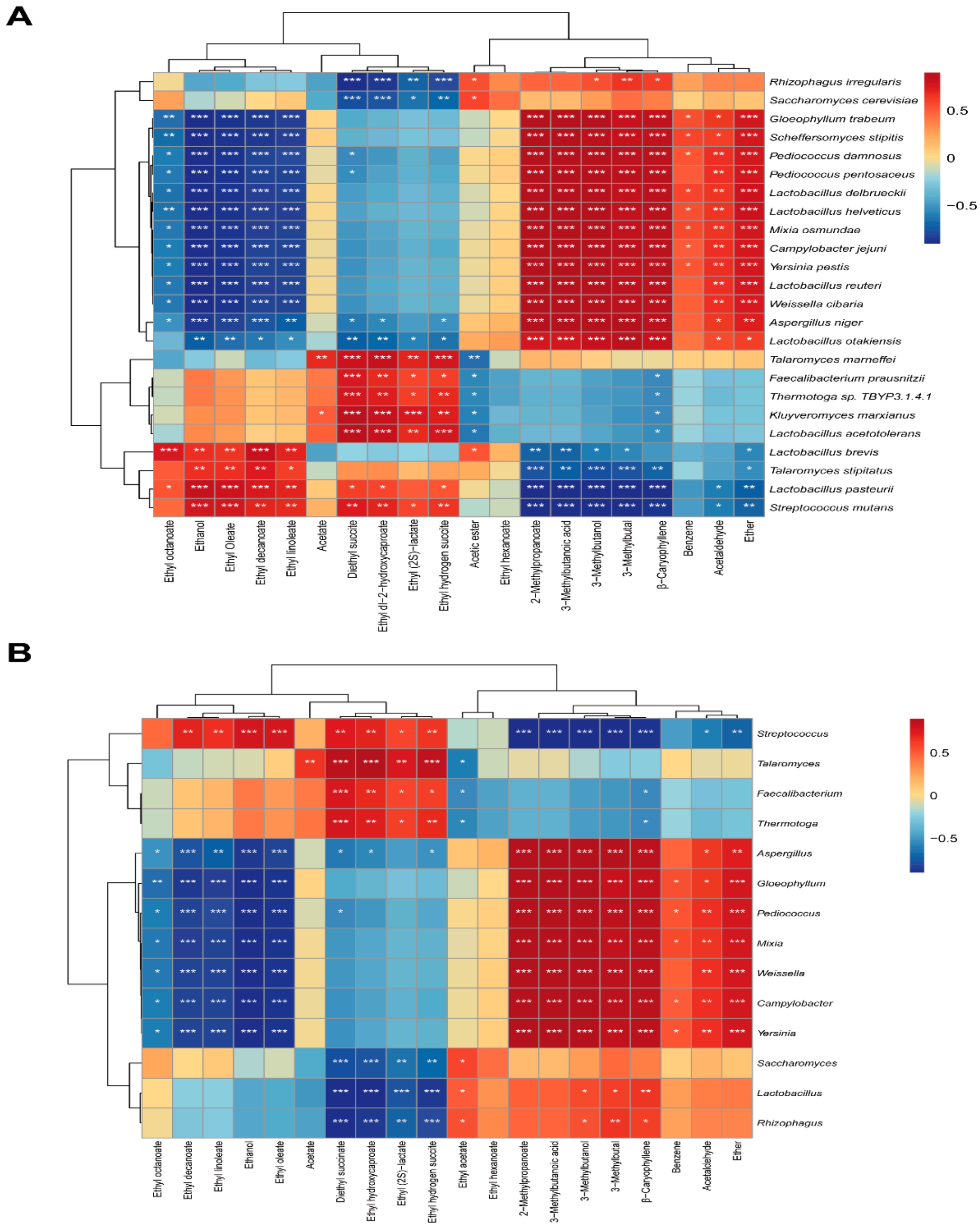

Figure S6. Correlation analysis between microbiota and volatile compounds by O2PLSDA model. (A) Statistical pearson's correlations between important genera (VIP > 1) and top 20 volatile compounds. (B) Statistical pearson's correlations between important species (VIP > 1) and top 20 volatile compounds. The correlated relationship with p-value < 0.05, 0.01, 0.001 was displayed by one, two and three asterisks, respectively.
